# Independent and joint impacts of high body mass index and aging on global burden of chronic kidney disease: insights from the Global Burden of Disease Study 2021

**DOI:** 10.3389/fnut.2025.1582534

**Published:** 2025-07-25

**Authors:** Yao Ma, Shun Chen, Yuanli Shen, Xiang Wang, Xinning Xie, Weihong Zhao

**Affiliations:** ^1^Division of Nephrology, Department of Geriatrics, Jiangsu Province Hospital and Nanjing Medical University First Affiliated Hospital, Nanjing, China; ^2^Department of Endocrinology, Diannan Central Hospital of Yunnan Province, Honghe First People’s Hospital, Mengzi, Yunnan, China; ^3^School of Public Health, Nanjing Medical University, Nanjing, China

**Keywords:** body mass index, aging, chronic kidney disease, deaths, disability-adjusted life years

## Abstract

**Introduction:**

We aimed to evaluate the levels and trends of CKD burden associated with high body mass index (BMI) from 1990 to 2021 and to investigate the role of aging.

**Methods:**

From the Global Burden of Disease Study 2021, we retrieved data and estimated CKD-related deaths, disability-adjusted life years (DALYs), age-standardized mortality rate (ASMR), and age-standardized DALYs rate (ASDR) attributable to high BMI by age, sex, socio-demographic index (SDI), and geographical regions. We calculated the estimated annual percentage changes (EAPCs) from 1990 to 2021 and projected attributable CKD burden through 2050. A cluster analysis was performed to identify changing patterns. We also used the decomposition analysis to evaluate the role of aging in observed trends.

**Results:**

Globally, high BMI was responsible for 418,402 CKD-related deaths and 1.04 million DALYs in 2021. The ASMR and ASDR were 5.06 and 122.08 per 100,000 population, showing an increasing trend from 1990 to 2021. The predicted results indicated that the attributable CKD burden will continue to rise through 2050. Males exhibited higher ASRs and EAPCs. Substantial geographic and regional disparities were observed, with an inverted “U”-shaped relationship between ASRs and SDI. With advancing age, the burden increased consistently, and sex differences varied. Decomposition analysis revealed that population aging was one contributing factor to the observed trends.

**Conclusion:**

Global CKD burden attributable to high BMI remains substantial. These findings underscore the urgency to address the growing public health challenge posed by obesity. Given the age, sex, and geographic differences, targeted strategies are needed.

## 1 Introduction

Chronic kidney disease (CKD) has emerged as a major and growing public health issue worldwide (1), associated with frequent cardiovascular events, progression to end-stage renal disease (ESRD), and elevated mortality risk (2). In 2019, CKD affected approximately 697 million individuals worldwide, leading to over 1.43 million deaths, with the figures continuing to rise annually (3). The growing burden places immense strain on healthcare systems, particularly in older adults, due in part to the aging of society. The natural decline in renal function with age makes older adults more susceptible to CKD (4, 5). At the same time, the coexistence of multiple chronic conditions further accelerates its progression, resulting in late-stage diagnoses. Given the detrimental impacts of CKD on prognosis, quality of life, and national economic resources, there is an urgency to develop optimized strategies for managing CKD and its risk factors.

Obesity is widely accepted as a significant risk factor for renal dysfunction. On one hand, obesity holds a tight link to type 2 diabetes mellitus and hypertension, both of which significantly contribute to ESRD development (6, 7). Moreover, the meta-analysis has indicated that after adjusting for these factors, obesity remains independently and positively associated with CKD risk (8). Multiple biological mechanisms link obesity with CKD, including altered renal hemodynamics, inflammation, lipid accumulation, and metabolic dysregulation (9). Epidemiologically, quantifying the disease burden associated with modifiable risk factors such as obesity is essential for understanding its relationship with CKD. However, CKD burden attributable to obesity, especially in epidemiological patterns and dynamic changes, remains unclear. Although several studies have reported the overall disease burden attributable to obesity (10, 11), they did not explore the spatiotemporal patterns of specific diseases, which may differ significantly.

To address this gap, we used the rich data from the Global Burden of Disease (GBD) Study 2021 to estimate the CKD burden attributable to high body mass index (BMI) globally and by subtypes in 2021. Additionally, we employed linear regression models to investigate spatiotemporal patterns and trends from 1990 to 2021, and constructed a Bayesian Age-Period-Cohort (BAPC) model to predict disease burden from 2022 to 2050. Furthermore, we examined differences across age groups and performed a decomposition analysis to assess the relative contribution of population aging to the observed trends. These findings aimed to inform healthcare professionals and policymakers in developing and implementing effective public health strategies.

## 2 Methods

### 2.1 Overview

The GBD 2021 dataset provides a comprehensive evaluation of health impacts associated with 369 diseases, injuries, and impairments, as well as 88 risk factors across 204 countries and territories (12, 13). Through the Global Health Data Exchange (GHDx) platform, we obtained the original data.^1^ In line with the Guidelines for Accurate and Transparent Health Estimates Reporting (GATHER) (14), data aggregation and analyses were performed.

We extracted annual data from 1990 to 2021 on CKD deaths, disability-adjusted life years (DALYs), age-standardized mortality rate (ASMR), and age-standardized DALYs rate (ASDR) attributable to high BMI, stratified by sex and age, for 204 countries and territories. These countries were categorized into 21 GBD regions based on epidemiological characteristics and geographic location. Social and economic development is considered to be tightly connected with health outcomes. The socio-demographic index (SDI) is calculated as the geometric mean of three factors: the total fertility rate for individuals under 25 years of age, the average educational attainment of the population aged 15 and older, and the lagged per capita income distribution. The SDI score ranges from 0 to 1, and accordingly, all countries and territories were grouped into five SDI quintiles (13).

### 2.2 Definition of CKD and high BMI

CKD is defined as renal dysfunction based on glomerular filtration rate (GFR) and proteinuria criteria in the GBD 2021 study. This encompasses an estimated GFR of less than 60 mL/min/1.73m^2^, calculated using serum creatinine, and/or an albumin-to-creatinine ratio (ACR) greater than 30 mg/g. A BMI of ≥25 kg/m^2^ is classified as high BMI in adults aged 20 years and older.

### 2.3 Estimation of high BMI-attributed CKD burden

The specific approach to estimate disease burden attributable to risk factors has been detailed elsewhere (12, 13). A comparative risk assessment framework was applied to estimate the attributable CKD burden. The steps were as follows. The first was determining relative risks associated with high BMI. Systematic reviews and meta-regression analyses were used to summarize the relative risks for the high BMI-CKD outcome pair as a function of exposure. Then, high BMI exposure levels and distributions were determined by age, sex, location, and year. Bayesian meta-regression models or spatiotemporal Gaussian process regression models were used to model these exposure levels and identify the theoretical minimum risk exposure level, which was defined as the level associated with the lowest observed risk in published trials and cohort studies. Finally, we calculated the population attributable fractions (PAFs) based on age, sex, location, and year to quantify attributable CKD burden. The detailed standard GBD PAF equation has been described in the prior study (15).

### 2.4 Statistical analysis

Globally and within subgroups stratified by sex, SDI, and geographical location, we calculated the counts and ASRs of deaths and DALYs to quantify CKD burden and the proportion attributable to high BMI. For each metric, we calculated 95% uncertainty intervals (UIs) derived from the 2.5th and 97.5th percentiles of 500 sampled level estimates (13). To analyze temporal trends, we used linear regression models to calculate the estimated annual percentage changes (EAPCs) in ASRs (16). The statistical significance of changes in ASRs is determined based on whether the 95% confidence interval (CI) of EAPC includes zero. Using EAPC values for ASMRs and ASDRs, we performed a hierarchical cluster analysis to categorize GBD regions (including World Bank classes) into four groups based on changing patterns in disease burden: significant increase, moderate increase, minor increase, and remained stable/decrease. Regional disparities were visualized through global maps and comparative analyses. We employed the ‘INLA’ and ‘BAPC’ packages in R to construct the BAPC model, which allowed us to predict the CKD burden attributable to high BMI through 2050. Smoothing spline models were applied at different levels to depict the relationship between SDI and high BMI-attributed CKD burden.

To explore the role of aging, adults aged 25 to 94 years were grouped in 5-year intervals, with the final group consisting of individuals aged 95 years and older. For each group, we calculated the numbers of deaths and DALYs, along with their ASRs, assessing the changes over time and across SDI regions. Additionally, to determine the impact of population aging, we used decomposition analysis to decompose the high BMI-attributed CKD burden by population age structure, population growth, and epidemiological changes (17). All statistical analyses were conducted using R 4.4.1.

## 3 Results

### 3.1 Global CKD burden and high BMI-attributed CKD burden in 2021

Global number of CKD-related deaths was 1,527,639 (95% UI: 1,389,377 to 1,638,914) in 2021, with corresponding DALYs reaching 44,453,684 (95% UI: 40,840,762 to 48,508,462). The ASMR and ASDR were 18.50 (95% UI: 16.72 to 19.85) and 529.62 (95% UI: 486.25 to 577.42) per 100,000 population, respectively. Males had higher numbers of deaths and DALYs, along with ASRs. The highest numbers of deaths (513,051) and DALYs (15,700,568) were found in middle SDI region, whereas low SDI region exhibited the highest ASRs (Supplementary Table 1).

**Figure 1:**
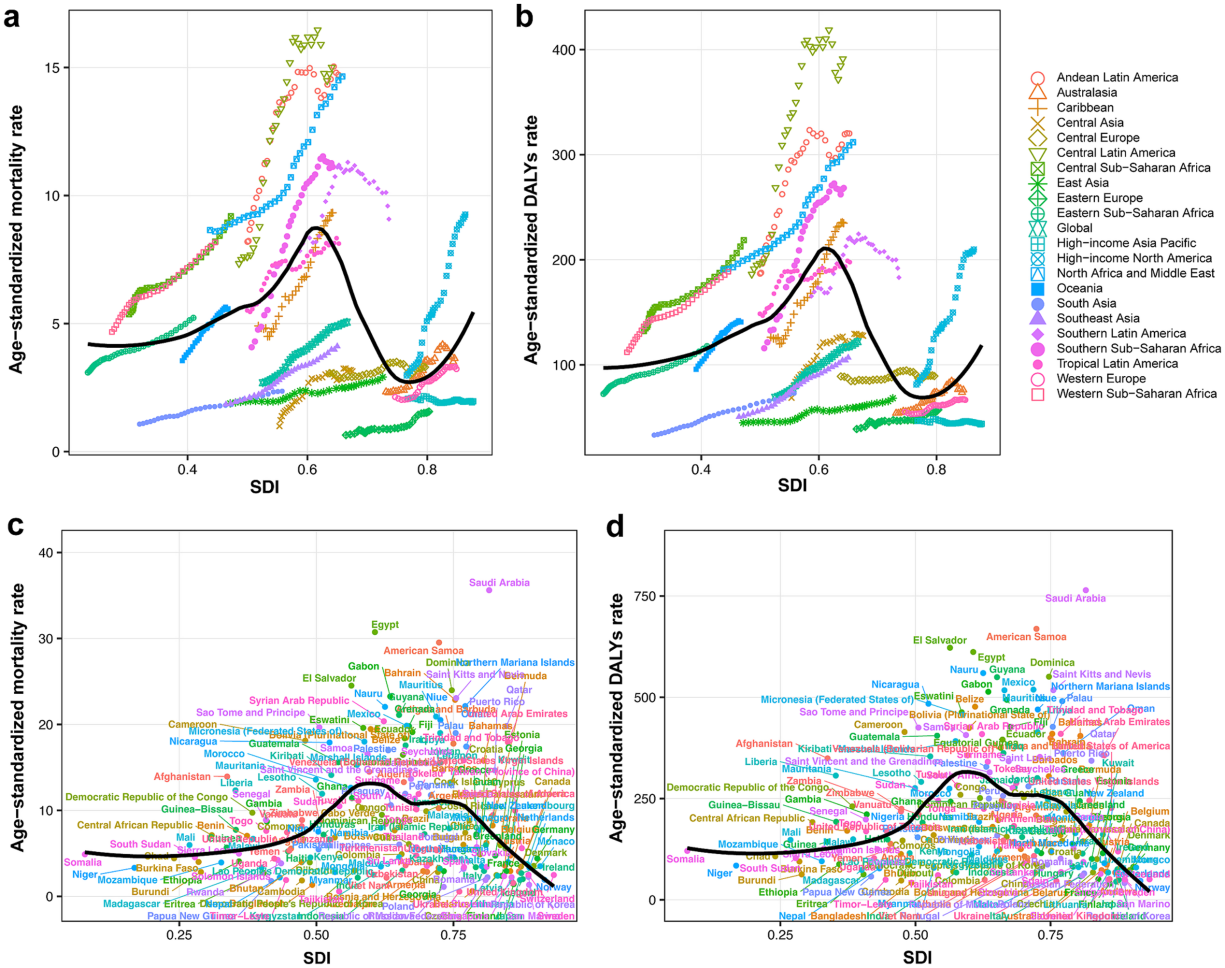
**(a)** CKD-related ASMR attributable to high BMI across 21 GBD regions by SDI, from 1990 to 2021. **(b)** CKD-related ASDR attributable to high BMI across 21 GBD regions by SDI, from 1990 to 2021. **(c)** CKD-related ASMR attributable to high BMI across 204 countries and territories by SDI in 2021. **(d)** CKD-related ASDR attributable to high BMI across 204 countries and territories by SDI in 2021. CKD, chronic kidney disease; ASMR, age-standardized mortality rate; BMI, body mass index; SDI, socio-demographic index; DALY, disability-adjusted life year; ASDR, age-standardized DALYs rate.

Globally, high BMI was responsible for an estimated 418,402 (95% UI: 224,309 to 621,353) CKD deaths, accounting for 27.4% of CKD-related deaths, with an ASMR of 5.06 (95% UI: 2.70 to 7.51). Corresponding DALYs were 10,422,561 (95% UI: 5,658,159 to 15,387,254), representing 23.4% of CKD-related DALYs, and the ASDR was 123.86 (95% UI: 67.23 to 182.96). While the absolute numbers of deaths and DALYs were higher in females, corresponding ASRs were higher among males. At the level of SDI regions, numbers of deaths and DALYs were highest in middle SDI region and lowest in low SDI region. ASMR and ASDR peaked in middle SDI and low-middle SDI regions, respectively, while values for these two metrics were the lowest in high-middle SDI region (Table 1). Smoothing spline analyses indicated consistent patterns between SDI levels and high BMI-attributed CKD burden on national and regional scales. An inverted “U”-shaped relationship between ASRs (for both deaths and DALYs) and SDI was observed. ASRs increased with SDI values below 0.6. However, in high-income North America, ASRs increased with rising SDI (Figure 1).

**Table 1:** Deaths and DALYs of chronic kidney disease attributable to high BMI in 1990 and 2021, and their estimated annual percentage changes from 1990 to 2021.

Characteristics	Deaths					DALYs				
	Number (thousand), 1990	ASMR (per 100,000 population), 1990	Number (thousand), 2021	ASMR (per 100,000 population), 2021	EAPC, 1990–2021	Number (thousand), 1990	ASDR (per 100,000 population), 1990	Number (thousand), 2021	ASDR (per 100,000 population), 2021	EAPC, 1990–2021
Global	91.99(47.20 to 140.78)	2.69 (1.37 to 4.14)	418.40 (224.31 to 621.35)	5.06 (2.70 to 7.51)	2.25 (2.13 to 2.36)	2670.09 (1367.92 to 4086.23)	69.13 (35.06 to 106.00)	10422.56 (5658.16 to 15387.25)	122.08 (66.25 to 180.18)	1.98 (1.89 to 2.07)
Sex										
Males	42.2(20.96 to 67.89)	2.96 (1.46 to 4.82)	195.61 (103.24 to 295.37)	5.47 (2.87 to 8.30)	2.24 (2.14 to 2.34)	1267.96 (636.25 to 1986.53)	72.50 (36.00 to 114.70)	5063.49 (2711.39 to 7531.65)	128.60 (68.10 to 190.90)	2.05 (1.96 to 2.13)
Females	49.78 (25.74 to 75.30)	2.54 (1.32 to 3.84)	222.79 (118.87 to 327.41)	4.77 (2.55 to 7.01)	2.21 (2.07 to 2.34)	1402.12 (726.93 to 2114.22)	67.04 (34.78 to 101.19)	5359.07 (2921.58 to 7834.93)	116.98 (63.78 to 170.94)	1.90 (1.80 to 2.00)
SDI										
High SDI	26.66 (13.93 to 39.25)	2.43 (1.27 to 3.58)	121.23 (63.49 to 179.47)	5.06 (2.75 to 7.39)	2.75 (2.60 to 2.90)	704.47 (368.72 to 1028.33)	64.68 (33.97 to 94.57)	2461.01 (1365.31 to 3490.31)	119.83 (68.69 to 167.37)	2.26 (2.15 to 2.38)
High-middle SDI	21.04 (10.95 to 32.11)	2.52 (1.31 to 3.87)	73.55 (39.50 to 110.23)	3.84 (2.06 to 5.76)	1.48 (1.40 to 1.56)	614.30 (316.14 to 936.79)	65.11 (33.26 to 99.79)	1724.60 (925.55 to 2563.74)	89.04 (47.48 to 132.50)	1.09 (1.02 to 1.16)
Middle SDI	25.29 (12.77 to 40.49)	3.02 (1.48 to 4.92)	134.84 (72.23 to 204.35)	5.49 (2.92 to 8.35)	2.08 (1.90 to 2.27)	775.65 (391.73 to 1247.67)	74.99 (37.30 to 120.40)	3626.44 (1961.57 to 5392.07)	135.25 (72.54 to 202.01)	2.03 (1.84 to 2.21)
Low-middle SDI	13.33 (6.89 to 21.13)	2.59 (1.31 to 4.15)	69.37 (36.96 to 104.79)	5.31 (2.78 to 8.04)	2.46 (2.39 to 2.53)	403.77 (208.02 to 634.94)	65.55 (33.75 to 104.30)	2013.41 (1064.39 to 3012.74)	136.22 (71.43 to 204.33)	2.50 (2.43 to 2.57)
Low SDI	5.52 (2.78 to 9.32)	2.84 (1.39 to 4.8)	18.98 (9.37 to 30.90)	4.31 (2.09 to 6.98)	1.30 (1.18 to 1.42)	167.82 (83.82 to 281.19)	71.68 (35.64 to 119.85)	586.37 (292.06 to 940.09)	107.94 (52.88 to 174.77)	1.24 (1.15 to 1.33)
GBD region										
Andean Latin America	1.54 (0.79 to 2.27)	8.2 (4.14 to 12.2)	8.42 (4.74 to 12.29)	14.72 (8.23 to 21.55)	1.88 (1.53 to 2.23)	38.54 (20.58 to 55.19)	186.82 (99.35 to 269.38)	189.67 (111.88 to 269.06)	320.22 (188.47 to 454.36)	1.71 (1.39 to 2.03)
Australasia	0.51 (0.26 to 0.80)	2.34 (1.18 to 3.62)	2.30 (1.23 to 3.40)	3.64 (1.98 to 5.35)	2.07 (1.81 to 2.34)	12.32 (6.42 to 18.56)	53.97 (28.18 to 81.36)	42.60 (23.91 to 60.10)	76.16 (43.49 to 106.96)	1.48 (1.29 to 1.66)
Caribbean	1.19 (0.64 to 1.80)	4.91 (2.63 to 7.54)	5.08 (2.81 to 7.59)	9.33 (5.17 to 13.92)	2.68 (2.49 to 2.86)	33.16 (18.16 to 47.97)	126.29 (69.09 to 183.86)	126.00 (72.50 to 182.33)	234.81 (135.4 to 339.31)	2.55 (2.39 to 2.71)
Central Asia	0.46 (0.23 to 0.74)	1.00 (0.50 to 1.60)	2.43 (1.23 to 3.84)	3.14 (1.58 to 5.02)	3.31 (2.82 to 3.81)	33.13 (17.92 to 49.69)	68.68 (37.39 to 102.79)	109.84 (57.97 to 166.72)	127.7 (67.69 to 193.21)	1.84 (1.57 to 2.11)
Central Europe	4.27 (2.34 to 6.40)	3.06 (1.68 to 4.58)	7.75 (4.30 to 11.53)	3.30 (1.82 to 4.91)	0.40 (0.21 to 0.59)	129.37 (71.07 to 190.55)	89.00 (48.98 to 131.40)	193.12 (107.52 to 281.85)	89.32 (50.24 to 130.68)	0.17 (0.05 to 0.29)
Central Latin America	5.43 (2.82 to 8.27)	7.34 (3.78 to 11.23)	37.00 (21.16 to 53.50)	15.01 (8.52 to 21.75)	2.78 (2.25 to 3.31)	160.41 (84.34 to 240.90)	186.21 (97.63 to 279.02)	1000.70 (584.01 to 1445.87)	391.24 (227.14 to 565.48)	2.78 (2.28 to 3.29)
Central Sub-Saharan Africa	0.98 (0.49 to 1.65)	5.36 (2.65 to 9.06)	4.08 (1.99 to 7.10)	9.19 (4.50 to 16.02)	1.57 (1.44 to 1.70)	30.77 (15.41 to 50.94)	132.20 (66.32 to 219.03)	127.98 (63.57 to 217.36)	218.67 (108.96 to 371.98)	1.45 (1.34 to 1.56)
East Asia	12.46 (5.68 to 23.61)	1.89 (0.86 to 3.56)	57.53 (28.92 to 96.87)	2.91 (1.44 to 4.88)	1.38 (1.28 to 1.48)	371.39 (166.75 to 709.82)	44.84 (20.23 to 85.00)	1459.58 (735.85 to 2378.61)	68.24 (34.25 to 112.21)	1.42 (1.30 to 1.54)
Eastern Europe	1.76 (0.91 to 2.74)	0.65 (0.33 to 1.00)	5.57 (2.97 to 8.29)	1.57 (0.84 to 2.33)	2.65 (2.28 to 3.03)	104.20 (55.03 to 156.16)	38.91 (20.31 to 58.48)	195.89 (111.94 to 283.09)	57.20 (32.55 to 82.88)	0.95 (0.82 to 1.09)
Eastern Sub-Saharan Africa	1.97 (0.93 to 3.50)	3.09 (1.39 to 5.42)	7.40 (3.54 to 12.37)	5.22 (2.44 to 8.76)	1.56 (1.49 to 1.64)	55.26 (25.06 to 95.10)	72.37 (33.49 to 127.01)	206.44 (99.74 to 337.03)	117.81 (56.44 to 195.00)	1.43 (1.36 to 1.49)
High-income Asia Pacific	3.64 (1.75 to 5.87)	2.07 (0.98 to 3.39)	12.98 (6.10 to 21.29)	1.97 (0.93 to 3.19)	−0.2 (−0.31 to −0.09)	90.15 (44.36 to 146.54)	46.60 (22.80 to 76.07)	224.78 (106.38 to 364.72)	43.85 (21.26 to 70.04)	−0.16 (−0.25 to −0.07)
High-income North America	10.81 (5.81 to 15.05)	2.97 (1.61 to 4.13)	65.22 (35.05 to 93.60)	9.26 (5.07 to 13.12)	4.09 (3.87 to 4.30)	282.08 (154.43 to 391.82)	81.26 (44.79 to 112.20)	1337.87 (769.24 to 1845.88)	209.80 (123.68 to 284.46)	3.40 (3.20 to 3.60)
North Africa and Middle East	11.91 (6.42 to 18.48)	8.65 (4.59 to 13.71)	56.21 (31.39 to 81.08)	14.65 (7.98 to 21.36)	1.86 (1.71 to 2.01)	315.24 (175.79 to 483.48)	190.68 (104.68 to 295.37)	1408.67 (811.44 to 2033.50)	311.83 (178.63 to 449.15)	1.72 (1.63 to 1.80)
Oceania	0.09 (0.04 to 0.15)	3.55 (1.60 to 6.38)	0.35 (0.15 to 0.59)	5.58 (2.51 to 9.51)	1.35 (1.21 to 1.49)	2.99 (1.40 to 5.06)	95.79 (43.85 to 164.39)	11.00 (4.92 to 17.90)	140.73 (62.80 to 233.05)	1.17 (1.03 to 1.30)
South Asia	5.58 (2.83 to 9.53)	1.07 (0.53 to 1.85)	32.71 (16.89 to 53.59)	2.36 (1.20 to 3.93)	2.55 (2.47 to 2.62)	205.94 (100.66 to 348.11)	33.15 (16.14 to 55.66)	1135.83 (572.35 to 1826.42)	72.97 (36.46 to 118.14)	2.65 (2.59 to 2.71)
Southeast Asia	4.33 (1.97 to 7.33)	1.85 (0.86 to 3.16)	25.02 (11.36 to 41.26)	4.11 (1.82 to 6.87)	2.66 (2.57 to 2.75)	137.99 (60.98 to 236.18)	50.45 (22.7 to 86.09)	732.32 (335.71 to 1219.01)	106.92 (48.70 to 176.80)	2.54 (2.44 to 2.64)
Southern Latin America	3.38 (1.79 to 4.90)	7.84 (4.14 to 11.42)	8.27 (4.56 to 12.19)	9.08 (5.03 to 13.35)	0.73 (0.37 to 1.10)	75.16 (41.35 to 106.86)	165.38 (90.15 to 235.43)	160.81 (90.31 to 230.30)	183.14 (103.23 to 260.25)	0.58 (0.26 to 0.90)
Southern Sub-Saharan Africa	0.99 (0.49 to 1.64)	4.08 (1.99 to 6.79)	5.47 (2.81 to 8.44)	11.12 (5.60 to 17.17)	3.25 (2.80 to 3.70)	33.50 (17.24 to 52.62)	115.81 (59.71 to 183.72)	158.45 (84.64 to 239.42)	268.33 (140.76 to 408.08)	2.73 (2.35 to 3.11)
Tropical Latin America	4.50 (2.48 to 6.49)	5.51 (2.98 to 8.07)	20.29 (11.57 to 27.90)	8.12 (4.61 to 11.22)	1.28 (1.09 to 1.47)	141.15 (78.38 to 201.58)	148.61 (81.45 to 214.84)	509.00 (293.91 to 689.73)	197.79 (113.71 to 269.46)	0.83 (0.64 to 1.02)
Western Europe	12.77 (6.41 to 19.41)	2.13 (1.07 to 3.26)	41.28 (19.85 to 64.19)	3.23 (1.56 to 4.98)	1.85 (1.68 to 2.03)	318.61 (161.10 to 479.30)	55.17 (27.82 to 82.99)	702.64 (345.13 to 1050.97)	66.64 (33.4 to 99.21)	0.84 (0.75 to 0.93)
Western Sub-Saharan Africa	3.42 (1.65 to 5.54)	4.67 (2.24 to 7.61)	13.05 (6.41 to 20.61)	8.17 (3.94 to 12.9)	1.66 (1.58 to 1.73)	98.75 (49.14 to 159.82)	111.99 (54.7 to 181.56)	389.38 (199.60 to 615.27)	188.72 (94.72 to 297.39)	1.56 (1.48 to 1.63)

DALY, disability-adjusted life year; BMI, body mass index; ASMR, age-standardized mortality rate; EAPC, estimated annual percentage change; ASDR, age-standardized DALYs rate; SDI, socio-demographic index.

Among GBD regions, the highest ASMR of CKD attributable to high BMI was found in Central Latin America (15.01 per 100,000 population; 95% UI: 8.52 to 21.75), and Eastern Europe reported the lowest ASMR (1.57 per 100,000 population; 95% UI: 0.84 to 2.33). The highest ASDR was observed in Central Latin America (391.24 per 100,000 population; 95% UI: 227.14 to 565.48), while high-income Asia Pacific had the lowest ASDR (43.85 per 100,000 population; 95% UI: 21.26 to 70.04) (Table 1).

**Figure 2:**
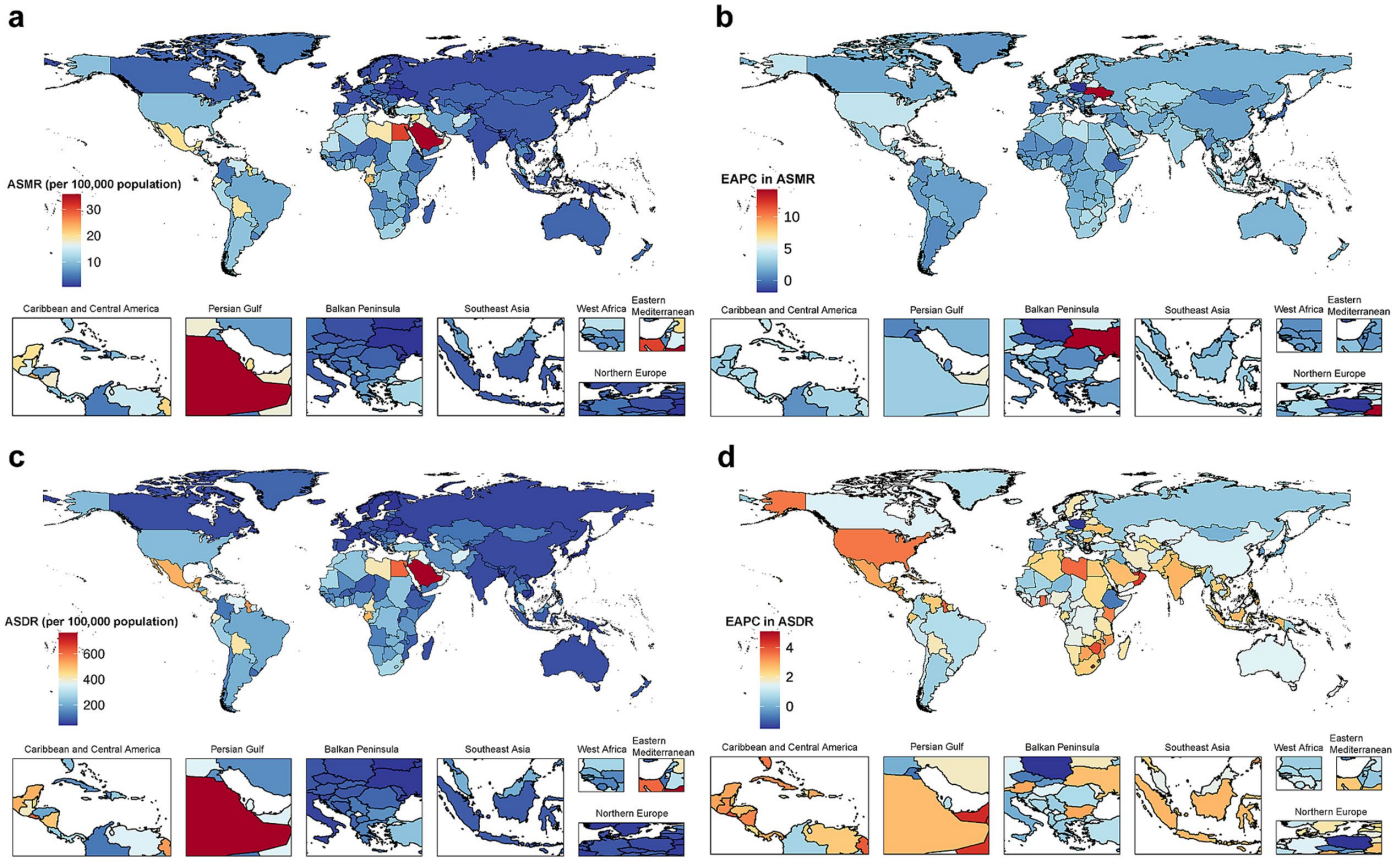
The global disease burden of CKD attributable to high BMI for both sexes combined in 204 countries and territories. **(a)** ASMR in 2021. **(b)** EAPC in ASMR from 1990 to 2021. **(c)** ASDR in 2021. **(d)** EAPC in ASDR from 1990 to 2021. CKD, chronic kidney disease; BMI, body mass index; ASMR, age-standardized mortality rate; EAPC, estimated annual percentage change; ASDR, age-standardized disability-adjusted life years rate.

Among all countries and territories analyzed, the high BMI-attributed CKD burden varied (Figures 2a,c). Saudi Arabia had the highest ASMR (35.62 per 100,000 population; 95% UI: 19.80 to 53.06), followed by Egypt and American Samoa. Ukraine had the lowest ASMR (0.56 per 100,000 population; 95% UI: 0.27 to 0.96), followed by Tajikistan and Belarus (Supplementary Table 2). Similarly, Saudi Arabia had the highest ASDR (764.26 per 100,000 population; 95% UI: 444.94 to 1126.37), and American Samoa and El Salvador followed, while the lowest ASDR was observed in Finland (39.78 per 100,000 population; 95% UI: 19.59 to 61.12), and the Republic of Korea and Belarus followed (Supplementary Table 3).

### 3.2 Temporal trends and cluster analysis of CKD burden attributable to high BMI from 1990 to 2021

Global CKD burden attributable to high BMI has shown a sustained upward trend over the study period. Compared to 1990, deaths and DALYs cases had increased approximately 3.5-fold and 2.9-fold in 2021, respectively. EAPC for ASMR was 2.25 (95% CI: 2.13 to 2.36), and EAPC for ASDR was 1.98 (95% CI: 1.89 to 2.07), indicating a faster growth rate compared to the overall CKD burden (Table 1 and Supplementary Table 1). Trends were similar across the sexes, with slightly higher EAPCs for males (Table 1).

Across all SDI regions, the attributable CKD burden consistently rose (Supplementary Figure 1). High SDI region showed the greatest increase in ASMR (EAPC = 2.75, 95% CI: 2.60 to 2.90), while low SDI region had the smallest (EAPC = 1.30, 95% CI: 1.18 to 1.42). For ASDR, the largest increase was found in low-middle SDI region (EAPC = 2.50, 95% CI: 2.43 to 2.57), and the smallest rise was in high-middle SDI region (EAPC = 1.09, 95% CI: 1.02 to 1.16) (Table 1).

Among GBD regions, trends in high BMI-attributed CKD burden varied (Table 1). We applied a hierarchical clustering analysis of the EAPC values for ASMR and ASDR to detect regions exhibiting similar trends. Results indicated that regions experiencing a significant increase in disease burden included high-income North America, North America, Southern Sub-Saharan Africa, Central Latin America, Southern Africa, America, and region of the Americas. Notably, the largest increases in ASMR and ASDR were found in high-income North America. ASRs remained stable or decreased in Southern Latin America, Central Europe, and high-income Asia Pacific (Supplementary Figure 2).

Among 204 countries and territories, ASMR remained stable or declined in 16 countries or territories. Poland exhibited the most significant decrease (EAPC = −1.89, 95% CI: −2.50 to −1.27). The highest increase in ASMR was seen in Ukraine (EAPC = 14.26, 95% CI: 12.24 to 16.31). For ASDR, 19 countries or territories showed stability or a decline, with Poland showing the most significant reduction (EAPC = −1.50, 95% CI: −1.89 to −1.11). Lesotho exhibited the largest rise in ASDR (EAPC = 5.10, 95% CI: 4.58 to 5.61). Additional details are available in Supplementary Tables 2, 3 and Figure 2.

### 3.3 Predicted trends from 2022 to 2050

**Figure 3:**
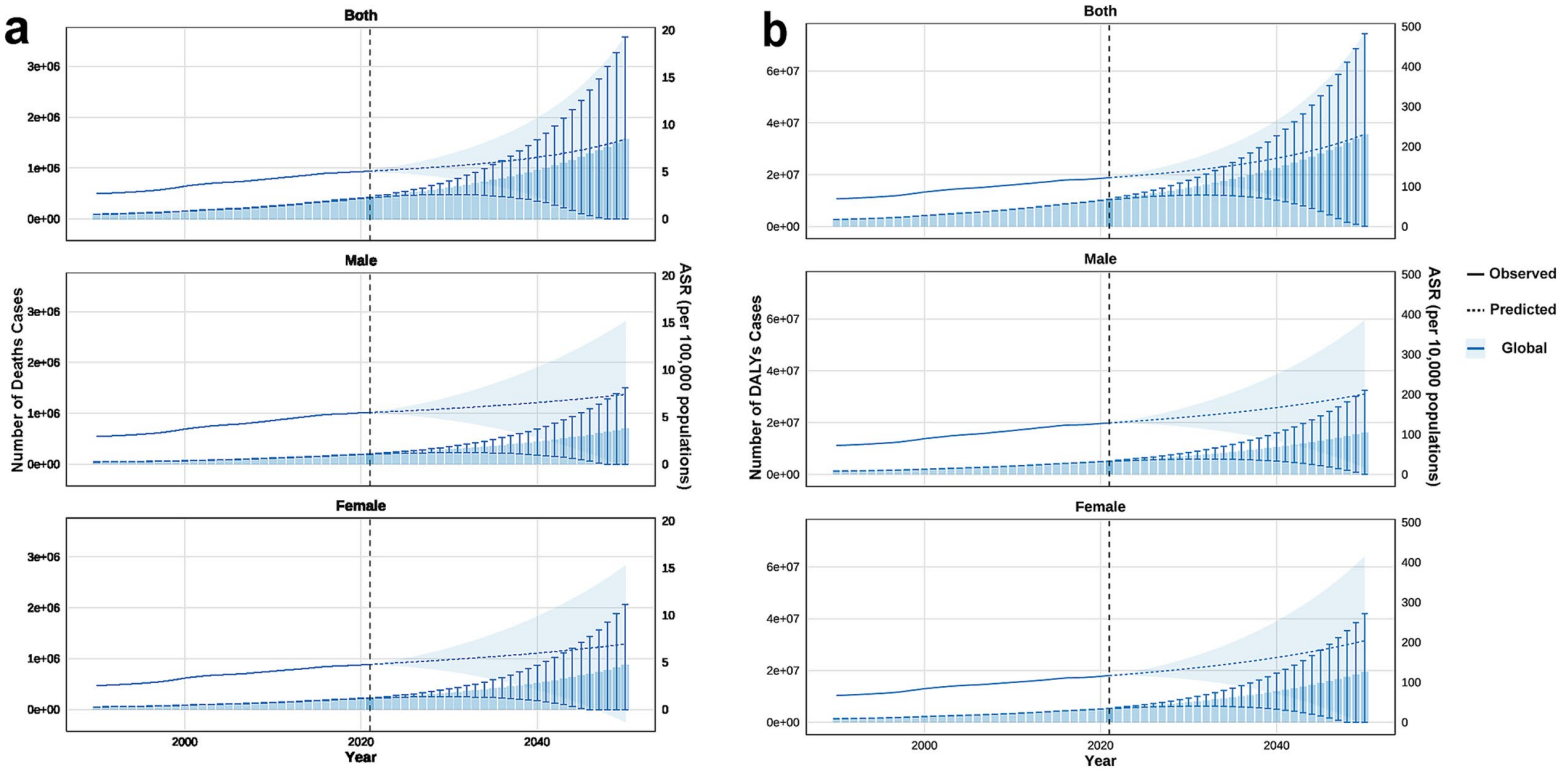
The predicted results in the CKD-related numbers and age-standardized rates of deaths and DALYs attributable to high BMI by sex globally, from 1990 to 2050. **(a)** Number and ASR of deaths. **(b)** Number and ASR of DALYs. CKD, chronic kidney disease; DALY, disability-adjusted life year; BMI, body mass index; ASR, age-standardized rate.

Projections indicated that from 2022 to 2050, the number of deaths and DALYs, along with their ASRs, will continue to increase. Although ASRs for males and females were expected to remain comparable, females will account for more cases (Figure 3).

### 3.4 Age-specific burden of CKD attributable to high BMI

**Figure 4:**
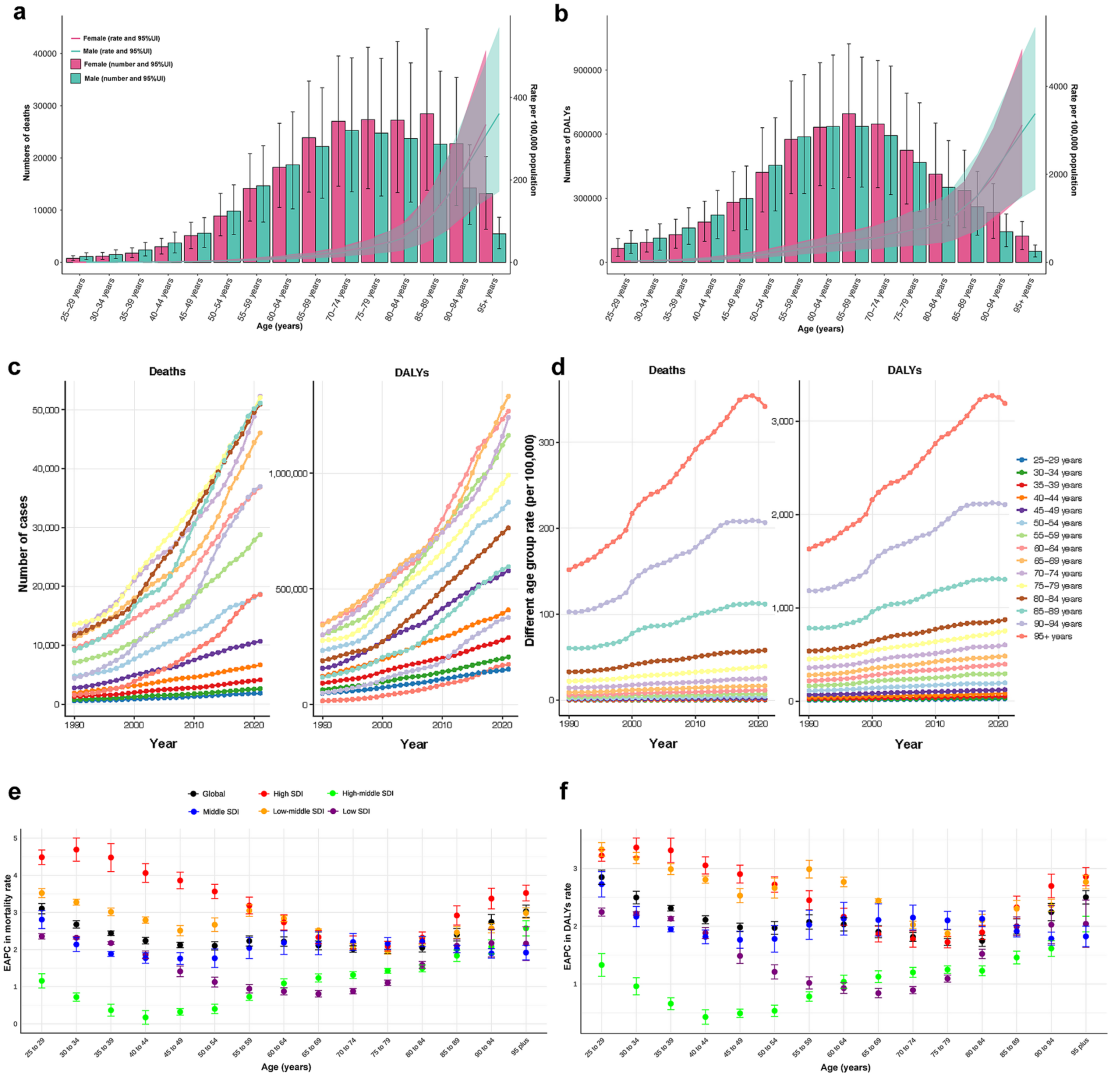
**(a)** Death numbers and rates of CKD attributable to high BMI by age and sex. **(b)** DALYs numbers and rates of CKD attributable to high BMI by age and sex. **(c)** Temporal trend of death and DALYs numbers attributable to high BMI by age, from 1990 to 2021. **(d)** Temporal trend of death and DALYs rates attributable to high BMI by age, from 1990 to 2021. **(e)** The age distribution of the trends in CKD-related death rate attributable to high BMI by SDI, from 1990 to 2021. **(f)** The age distribution of the trends in CKD-related DALYs rate attributable to high BMI by SDI, from 1990 to 2021. CKD, chronic kidney disease; BMI, body mass index; DALY, disability-adjusted life year; SDI, socio-demographic index.

The highest number of CKD deaths due to high BMI was observed in males aged 70–74 and females aged 85–89. Notably, before the age of 65, there were more male decedents than females. However, beyond 65 years, female deaths exceeded those of males, with the disparity widening progressively with age (Figure 4a). The distribution of DALYs cases followed an approximate normal curve, peaking in the age group of 60–64 for males and 65–69 for females. Similarly, DALYs cases were higher in males before the age of 65, while females exhibited higher cases in older adults (Figure 4b). Age-specific rates of CKD-related deaths and DALYs due to high BMI exhibited non-linear growth, with a marked acceleration after the age of 85 (Figures 4a,b).

Between 1990 and 2021, CKD-related deaths and DALYs cases attributable to high BMI were primarily observed among older adults and increased rapidly in this population, while remaining relatively stable among younger individuals (Figure 4c). Across all periods, the age-specific rates followed consistent patterns, peaking in individuals aged 95 years and older and showing a rapid annual increase until 2019 (Figure 4d).

The EAPCs for age-specific deaths and DALYs rates remained stable globally from 1990 to 2021. Among younger adults, EAPCs varied significantly across SDI regions, being highest in high SDI region and lowest in high-middle SDI region. However, with advancing age, the disparities tend to diminish (Figures 4e,f).

### 3.5 Decomposition analysis of disease burden

**Figure 5:**
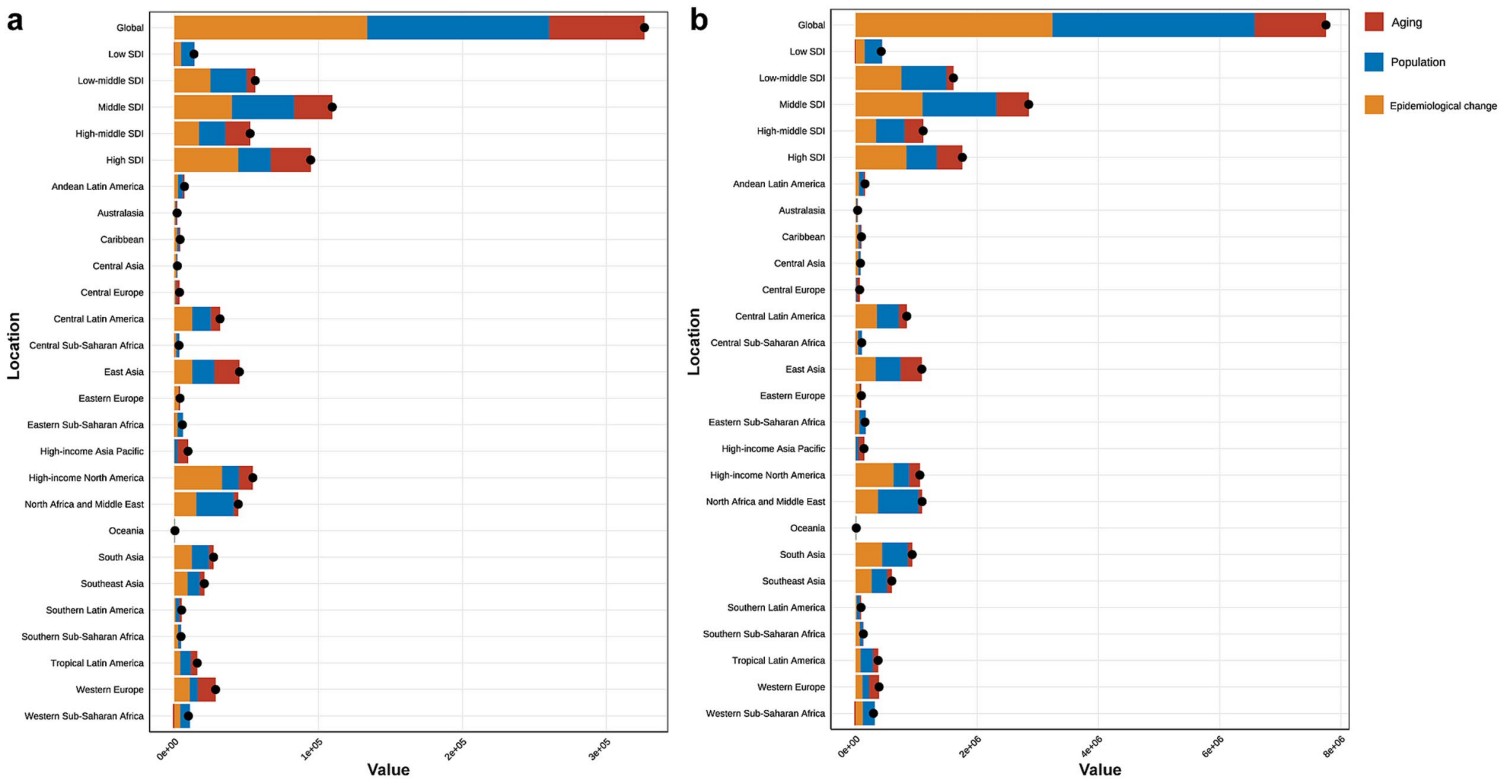
**(a)** Decomposition analysis of high BMI-attributed CKD burden change in mortality by SDI and 21 GBD region, from 1990 to 2021. **(b)** Decomposition analysis of high BMI-attributed CKD burden change in DALYs by SDI and 21 GBD region, from 1990 to 2021. BMI, body mass index; CKD, chronic kidney disease; SDI, socio-demographic index.

The decomposition analysis assessed the relative contributions of aging, population growth, and epidemiological changes to the rising disease burden from 1990 to 2021. The results revealed that all three factors contributed to the increasing burden. Aging played a relatively prominent role in the middle, high-middle, and high SDI regions. Among GBD regions, the rise in CKD burden due to high BMI in Central Europe, East Asia, high-income Asia Pacific, and Western Europe was primarily driven by aging (Figure 5).

## 4 Discussion

This study provides the first comprehensive analysis of the global CKD burden attributable to high BMI based on the GBD Study 2021 framework. From 1990 to 2021, while the overall CKD burden increased slowly, CKD-related deaths, DALYs, and corresponding ASRs attributable to high BMI exhibited a more pronounced and sustained growth. The burden increases with age and exhibits differences across sexes and regions. Population aging emerged as one critical driver of this trend, with projections indicating a continued rise from 2022 to 2050.

Since the first study linking obesity and kidney disease, a century has passed (18). Nowadays, the association between obesity and CKD is well established. Chronic inflammation is a hallmark of obesity-associated CKD, contributing to glomerulosclerosis, vascular damage, and fibrosis. Crosstalk between adipocytes and activated M1 macrophages maintains the inflammation status in adipose tissue, with activated macrophages releasing pro-inflammatory chemokines (19). Moreover, adipose tissue is considered more than just a fat storage depot but as an endocrine organ capable of secreting various adipokines (20). Among the obesity, upregulation of transforming growth factor-β1 (TGF-β1) in the kidney is induced by elevated leptin levels, promoting extracellular matrix accumulation, which leads to glomerular and tubular basement membrane thickening, glomerulosclerosis, and tubulointerstitial fibrosis (21). In contrast, adiponectin, which alleviates renal fibrosis, is reduced in obesity (22). Recent findings have also highlighted the possible impact of gastrointestinal dysbiosis and endothelial cell subtype specificity in CKD progression (23, 24). For CKD prevention and intervention, effective monitoring and management of modifiable risk factors represent cost-effective strategies. Despite robust evidence supporting the benefits of weight loss (25), a significant proportion of CKD patients remain obese or severely obese. Comprehensive health interventions and public awareness campaigns are urgently needed to address this growing issue.

Our results indicated that while the global CKD burden showed a slow upward trend, the burden attributable to high BMI grew more pronounced. Rising PAFs underscored the widespread prevalence of obesity and its detrimental effects on renal function. We also identified regional disparities across different levels of SDI. Low SDI region exhibited the highest CKD-related ASMR and ASDR in 2021, whereas middle and low-middle SDI regions had relatively higher high BMI-attributable CKD-related ASMR and ASDR, forming an inverted “U”-shaped association. The sociospatial inequalities of obesity prevalence, weight loss measures, CKD prevention strategies, and healthcare may contribute to this pattern. In middle and low-middle SDI regions, economic development and social changes have driven a shift toward more sedentary lifestyles and an increase in the consumption of high-fat, high-sugar diets, which have contributed to a rising obesity prevalence. In contrast, although low SDI regions exhibit certain unhealthy lifestyle factors, their lower levels of economic development often result in simpler dietary patterns, with reduced intake of high-fat and high-sugar foods. Residents in high SDI region benefit from better healthcare systems and more effective prevention strategies. However, it is critical to acknowledge that low-middle and low SDI regions often lack adequate healthcare facilities and advanced laboratory diagnostic services, leading to underdiagnosis and an underestimated burden of CKD. Despite being a high-SDI region, high-income North America experienced the most significant increases in ASMR and ASDR. Similar findings have been reported in previous studies. Among GBD regions, high-income North America exhibited the highest attributable proportions of age-standardized DALYs due to high BMI (26). Furthermore, high fasting plasma glucose-attributed CKD burden had grown substantially in this region (27). Some high-income countries may face resource constraints in prioritizing CKD prevention and addressing related risk factors such as diabetes, hypertension, and obesity. This may be due to reduced healthcare budget allocations and potentially insufficient awareness of CKD-related risk factors. Furthermore, the prevalent fast-food culture, sugary beverage consumption, and associated dietary patterns contribute to the higher burden of disease attributable to obesity in high-income North America. Collectively, our study highlighted significant transitions in the obesity and disease spectrum across regions with varying levels of development. To address this issue, a global action plan targeting obesity is essential, particularly focusing on regions with an SDI of around 0.6, which could serve as a strategic starting point.

Sex-specific analyses revealed a significant increase in high BMI-attributable CKD deaths and DALYs for both sexes, yet sex-specific differences varied by age. While females exhibited higher absolute numbers of deaths and DALYs, ASRs of these indicators were higher in males, indicating that the burden of CKD attributable to high BMI is more significant among older females. Age-stratified analyses supported this observation. Tsao et al. (28) reported similar findings that among participants aged over 45 years, the prevalence of renal impairment (defined as CKD stage >3 or proteinuria) was higher in obese females than in males (20.3% vs. 10.1%). Furthermore, Shahali et al. (29) demonstrated over a 12-year follow-up period that obese females faced a greater risk of a decline in eGFR exceeding 40%. As age increases, the progression of CKD is more rapid in obese women. The underlying mechanisms are complex, primarily involving changes in body composition and hormone levels. The visceral fat accumulation increases with age and is more pronounced in females, nearly quadrupling between 25 and 65 years old (30, 31). On the other hand, estrogen, which involves renoprotective effects (32), decreases with age. Non-biological factors also play a role, including socio-cultural influences, personal preferences, and disparities in access to care. For instance, older females are 2–3 times more likely to opt for conservative management over renal replacement therapy (RRT) than males when consulting nephrologists (33, 34). Additionally, a greater proportion of elderly women live alone, without caregivers, a role typically assumed by spouses. These factors contribute to the heavier CKD burden among obese older females together. These sex disparities underscore the necessity of developing sex-specific public health strategies to address the growing influence of high BMI.

A higher burden of age-specific deaths and DALYs was observed among older and middle-aged adults. Several factors may explain this pattern. First, the impact of obesity on renal function may differ across age groups. Lu et al. (35) revealed that obesity before the age of 40 was not significantly associated with a rapid decline in renal function. However, the risk increased with age and was highest among individuals aged over 80 years. The impact of obesity appears and becomes pronounced primarily due to age-related changes in kidney. Beginning at age 30, the number of nephrons begins to irreversibly decline (36). In earlier life stages, the remaining functional nephrons can compensate for the effects of abnormal BMI, allowing GFR to remain relatively stable even in individuals with extreme BMI. However, as age advances, particularly in older adulthood, the functional nephrons diminish, reducing compensatory ability. Obesity acts as a “secondary hit,” accelerating the decline in GFR. Second, older and middle-aged adults face unique age-related social challenges, including stress and loneliness (37, 38), which can negatively influence health behaviors and CKD management. Furthermore, despite experiencing health concerns, older adults often delay seeking medical care or preventive measures (39), resulting in later disease stages at diagnosis. Therefore, future interventions should prioritize strategies that promote physical and social engagement among older adults to enhance their overall health.

Recent advancements in antidiabetic pharmacotherapy have shown promising outcomes, with these therapeutic agents proving effective not only in glycemic control but also in weight management and renal protection (40, 41). Glucagon-like peptide-1 receptor agonists (GLP-1 RAs), approved by the US Food and Drug Administration (FDA) as weight loss medications, have demonstrated significant potential in managing obesity-related renal injury. GLP-1 exerts its hypoglycemic effect by stimulating insulin secretion and inhibiting glucagon release. Additionally, it delays gastric emptying, suppresses hunger, and ultimately contributes to weight reduction (42, 43). Regarding renal protection, GLP-1 RAs upregulate adenosine monophosphate kinase (AMPK) through cyclic adenosine monophosphate (cAMP) accumulation, thereby initiating immunoregulatory responses (44, 45). Furthermore, GLP-1 RAs have been shown to induce natriuresis and diuresis, subsequently activating tubuloglomerular feedback to reduce glomerular pressure. For patients who struggle to achieve weight loss through diet and exercise alone, GLP-1 RAs represent a promising therapeutic option.

This study has several limitations. First, the GBD study is an observational study and, as such, is unable to establish causal relationships. Second, body fat metrics other than BMI were not included. Although BMI is a convenient and widely used metric for assessing obesity, it has limitations in differentiating between fat and lean mass and cannot capture fat distribution. Third, imperfect healthcare systems in underdeveloped countries may result in misdiagnosis or underdiagnosis of CKD, resulting in an underestimation of cases. On the other hand, the lack of high-quality, population-based studies forces estimates to depend on data from higher geographical levels. Fourth, the diagnosis of CKD relies on eGFR, and the GFR estimating equations generally perform poorly when validated outside the populations in which they were developed (46), potentially introducing bias.

## 5 Conclusion

The global burden of CKD attributable to high BMI has shown a consistent upward trend from 1990 to 2021, with projections indicating a further increase by 2050. To effectively mitigate this growing burden, comprehensive strategies for the prevention, assessment, and management of CKD are of paramount importance. Given that obesity is a modifiable risk factor, targeted weight reduction policies and interventions tailored to specific age groups, sexes, and regional contexts should be encouraged in the future. Our findings provide valuable insights for policymakers in formulating evidence-based strategies to address this pressing public health challenge.

## Data Availability

The original contributions presented in the study are included in the article/Supplementary material, further inquiries can be directed to the corresponding author.

## Footnotes

^1^ https://ghdx.healthdata.org/
