# Supplementary material for: Independent and joint impacts of high body mass index and aging on global burden of chronic kidney disease: insights from the Global Burden of Disease Study 2021
Source: Front Nutr. 2025 Jul 25;12:1582534. doi: 10.3389/fnut.2025.1582534 (PMC12331487; doi:10.3389/fnut.2025.1582534)
Supplement: Supplementary file 1 [file Table_1.DOCX]

***Supplementary Materials***

**Contents**

**Supplementary Table 1.** Deaths and DALYs of chronic kidney disease in 1990 and 2021, and their estimated annual percentage changes from 1990 to 2021 2

**Supplementary Table 2.** Deaths of chronic kidney disease attributable to high BMI in 1990 and 2021, and the estimated annual percentage changes from 1990 to 2021 3

**Supplementary Table 3.** DALYs of chronic kidney disease attributable to high BMI in 1990 and 2021, and the estimated annual percentage changes from 1990 to 2021 9

**Supplementary Figure 1.** The global disease burden of CKD attributable to high BMI by SDI 15

**Supplementary Figure 2.** Results of cluster analysis based on the EAPC values for CKD-related ASMRs and ASDRs attributable to high BMI, from 1990 to 2021 16

**Supplementary Table 1. Deaths and DALYs of chronic kidney disease in 1990 and 2021, and their estimated annual percentage changes from 1990 to 2021.**

| **Characteristics** | **Deaths** | | | | | **DALYs** | | | | |
| --- | --- | --- | --- | --- | --- | --- | --- | --- | --- | --- |
|  | Number (thousand), 1990 | ASMR (per 100,000 population), 1990 | Number (thousand), 2021 | ASMR (per 100,000 population), 2021 | EAPC, 1990-2021 | Number (thousand), 1990 | ASDR (per 100,000 population), 1990 | Number (thousand), 2021 | ASDR (per 100,000 population), 2021 | EAPC, 1990-2021 |
| **Global** | 552.67  (513.46 to 607.92) | 14.85  (13.64 to 16.38) | 1527.64  (1389.38 to 1638.91) | 18.50  (16.72 to 19.85) | 0.82  (0.76 to 0.89) | 20739.90  (18843.68 to 22588.53) | 479.85  (439.18 to 523.79) | 44453.68  (40840.76 to 48508.46) | 529.62  (486.25 to 577.42) | 0.37  (0.33 to 0.41) |
| **Sex** |  |  |  |  |  |  |  |  |  |  |
| Male | 291.67  (259.31 to 334.44) | 18.13  (16.26 to 21.03) | 794.52  (719.35 to 856.33) | 21.91  (19.66 to 23.60) | 0.75  (0.69 to 0.80) | 11069.49  (9601.63 to 12296.84) | 546.41  (476.93 to 612.62) | 23759.75  (21473.24 to 26215.37) | 603.40  (546.08 to 663.35) | 0.39  (0.36 to 0.43) |
| Female | 261.01  (237.13 to 288.00) | 12.64  (11.44 to 13.99) | 733.12  (654.83 to 795.63) | 15.9  (14.22 to 17.27) | 0.83  (0.76 to 0.90) | 9670.41  (8788.28 to 10592.76) | 426.90  (389.12 to 467.43) | 20693.93  (18836.61 to 22728.83) | 465.69  (424.02 to 511.12) | 0.30  (0.25 to 0.35) |
| **SDI** |  |  |  |  |  |  |  |  |  |  |
| High SDI | 100.03  (92.11 to 104.19) | 9.22  (8.45 to 9.62) | 340.08  (289.02 to 369.67) | 14.11  (12.30 to 15.21) | 1.73  (1.61 to 1.86) | 2898.45  (2605.44 to 3169.02) | 277.64  (250.03 to 303.24) | 7115.74  (6464.11 to 7759.25) | 358.51  (324.74 to 390.20) | 1.07  (0.99 to 1.16) |
| High-middle SDI | 99.20  (91.76 to 110.67) | 11.36  (10.43 to 12.65) | 226.80  (201.67 to 252.70) | 12.02  (10.68 to 13.38) | 0.25  (0.16 to 0.34) | 3534.41  (3184.09 to 3954.98) | 360.84  (325.48 to 401.53) | 5944.28  (5372.85 to 6623.41) | 324.64  (293.58 to 360.92) | -0.35  (-0.42 to -0.28) |
| Middle SDI | 177.30  (162.78 to 198.07) | 19.07  (17.39 to 21.35) | 513.05  (458.87 to 556.75) | 20.89  (18.45 to 22.67) | 0.38  (0.29 to 0.48) | 7031.76  (6325.50 to 7794.73) | 585.56  (531.80 to 652.64) | 15700.57  (14206.92 to 17147.17) | 596.45  (540.33 to 650.48) | 0.12  (0.04 to 0.20) |
| Low-middle SDI | 64.56  (52.43 to 78.60) | 21.48  (17.64 to 27.68) | 171.02  (149.78 to 193.48) | 27.17  (23.84 to 31.41) | 0.79  (0.75 to 0.83) | 4690.10  (4056.13 to 5163.16) | 609.44  (545.07 to 686.29) | 10611.26  (9599.82 to 11771.76) | 686.98  (622.50 to 765.20) | 0.40  (0.38 to 0.42) |
| Low SDI | 64.66  (57.36 to 73.65) | 29.72  (26.31 to 34.62) | 136.80  (118.87 to 157.58) | 29.43  (26.13 to 33.79) | -0.08  (-0.20 to 0.03) | 2564.55  (2265.15 to 2870.45) | 853.94  (760.14 to 971.76) | 5042.41  (4408.65 to 5841.52) | 791.80  (704.14 to 909.10) | -0.34  (-0.42 to -0.25) |

Abbreviations: DALY, disability-adjusted life year; ASMR, age-standardized mortality rate; EAPC, estimated annual percentage change; ASDR, age-standardized DALYs rate; SDI, socio-demographic index.

**Supplementary Table 2.** **Deaths of chronic kidney disease** **attributable to high BMI in 1990 and 2021, and the estimated annual percentage changes from 1990 to 2021.**

| **Location** | **Number, 1990** | **ASMR, 1990** | **Number, 2021** | **ASMR, 2021** | **EAPC, 1990-2021** |
| --- | --- | --- | --- | --- | --- |
| Afghanistan | 627 (311 to 1068) | 9.41 (4.71 to 16.32) | 1219 (502 to 2329) | 13.94 (5.77 to 27.23) | 1.38 (1.19 to 1.58) |
| Albania | 60 (33 to 95) | 3.43 (1.9 to 5.45) | 159 (85 to 256) | 3.83 (2.02 to 6.11) | 0.68 (0.42 to 0.93) |
| Algeria | 531 (262 to 1013) | 6.28 (3.06 to 11.81) | 3542 (1891 to 5422) | 13.29 (7.13 to 20.68) | 2.91 (2.65 to 3.17) |
| American Samoa | 2 (1 to 3) | 10.46 (4.49 to 18.68) | 12 (6 to 20) | 29.53 (13.18 to 48.5) | 3.55 (3.29 to 3.82) |
| Andorra | 1 (1 to 3) | 3.38 (1.63 to 5.86) | 5 (2 to 9) | 2.89 (1.36 to 4.95) | -0.11 (-0.34 to 0.12) |
| Angola | 103 (44 to 190) | 3.07 (1.31 to 5.69) | 549 (243 to 1002) | 5.56 (2.44 to 10.06) | 1.71 (1.54 to 1.88) |
| Antigua and Barbuda | 5 (2 to 7) | 8.36 (4.41 to 13.43) | 18 (10 to 27) | 17.77 (9.94 to 27.28) | 3.11 (2.84 to 3.39) |
| Argentina | 2779 (1487 to 4044) | 9.21 (4.92 to 13.52) | 5963 (3349 to 8607) | 10.26 (5.77 to 14.83) | 0.59 (0.23 to 0.96) |
| Armenia | 5 (2 to 8) | 0.19 (0.09 to 0.3) | 124 (61 to 196) | 2.85 (1.41 to 4.54) | 8.7 (7.37 to 10.06) |
| Australia | 429 (212 to 671) | 2.36 (1.16 to 3.69) | 1930 (1020 to 2895) | 3.56 (1.9 to 5.31) | 2.04 (1.76 to 2.32) |
| Austria | 203 (102 to 300) | 1.65 (0.85 to 2.43) | 1154 (605 to 1703) | 4.76 (2.5 to 7.02) | 5.18 (4.5 to 5.87) |
| Azerbaijan | 67 (33 to 112) | 1.35 (0.66 to 2.2) | 286 (146 to 480) | 2.94 (1.48 to 5) | 2.94 (2.63 to 3.26) |
| Bahamas | 12 (7 to 18) | 8.14 (4.32 to 11.75) | 61 (34 to 89) | 15.79 (8.59 to 23.31) | 2.79 (2.55 to 3.03) |
| Bahrain | 13 (7 to 22) | 10.86 (5.37 to 19.36) | 115 (70 to 167) | 22.87 (13.17 to 34.3) | 2.12 (1.75 to 2.49) |
| Bangladesh | 343 (151 to 636) | 0.78 (0.34 to 1.41) | 1689 (826 to 3001) | 1.34 (0.65 to 2.36) | 2.03 (1.85 to 2.21) |
| Barbados | 21 (11 to 31) | 7.08 (3.96 to 10.49) | 68 (38 to 105) | 13.29 (7.49 to 20.26) | 2.6 (2.28 to 2.92) |
| Belarus | 17 (8 to 27) | 0.14 (0.06 to 0.22) | 97 (49 to 152) | 0.61 (0.3 to 0.95) | 4.81 (3.92 to 5.71) |
| Belgium | 348 (177 to 533) | 2.19 (1.12 to 3.32) | 940 (455 to 1419) | 2.94 (1.44 to 4.41) | 1.59 (1.29 to 1.89) |
| Belize | 8 (4 to 11) | 8.57 (4.54 to 12.44) | 56 (33 to 79) | 19.45 (11 to 28.46) | 3.12 (2.61 to 3.63) |
| Benin | 81 (36 to 134) | 4.41 (1.91 to 7.36) | 323 (147 to 537) | 7.04 (3.19 to 11.78) | 1.43 (1.34 to 1.53) |
| Bermuda | 4 (2 to 6) | 6.97 (3.96 to 9.5) | 12 (7 to 17) | 8.25 (4.83 to 11.57) | 1.06 (0.75 to 1.37) |
| Bhutan | 6 (3 to 11) | 2.78 (1.25 to 4.89) | 23 (11 to 41) | 3.98 (1.84 to 7.12) | 1.22 (1.17 to 1.27) |
| Bolivia (Plurinational State of) | 297 (144 to 471) | 10.31 (4.95 to 16.76) | 1602 (842 to 2532) | 19.46 (9.84 to 30.72) | 2.23 (2.14 to 2.33) |
| Bosnia and Herzegovina | 86 (45 to 139) | 2.38 (1.24 to 3.83) | 212 (104 to 349) | 3.31 (1.62 to 5.46) | 1.02 (0.81 to 1.22) |
| Botswana | 13 (6 to 24) | 2.89 (1.26 to 5.32) | 89 (43 to 148) | 7.68 (3.62 to 12.82) | 3.61 (3.31 to 3.9) |
| Brazil | 4375 (2404 to 6346) | 5.5 (2.97 to 8.07) | 19702 (11197 to 27199) | 8.07 (4.55 to 11.2) | 1.26 (1.06 to 1.45) |
| Brunei Darussalam | 4 (2 to 6) | 4.36 (2.02 to 7.63) | 17 (9 to 27) | 6.22 (3.08 to 10.37) | 1.71 (1.49 to 1.93) |
| Bulgaria | 238 (131 to 348) | 2.4 (1.31 to 3.5) | 859 (453 to 1316) | 6 (3.22 to 9.17) | 3.79 (3.32 to 4.27) |
| Burkina Faso | 99 (43 to 176) | 2.71 (1.19 to 4.89) | 321 (139 to 577) | 4 (1.75 to 7.11) | 1.31 (1.23 to 1.39) |
| Burundi | 46 (21 to 85) | 2.22 (1.03 to 4.17) | 111 (51 to 209) | 2.84 (1.28 to 5.24) | 0.32 (0.14 to 0.5) |
| Cabo Verde | 5 (2 to 8) | 2.21 (1.01 to 3.6) | 30 (13 to 51) | 6.85 (3 to 12.02) | 3.46 (3.2 to 3.71) |
| Cambodia | 52 (22 to 97) | 1.27 (0.54 to 2.38) | 206 (82 to 373) | 1.85 (0.74 to 3.34) | 1.12 (0.87 to 1.37) |
| Cameroon | 441 (203 to 698) | 11.96 (5.36 to 19.33) | 1869 (942 to 3212) | 18.12 (8.99 to 30.7) | 0.79 (0.55 to 1.04) |
| Canada | 743 (375 to 1094) | 2.34 (1.18 to 3.45) | 2891 (1398 to 4368) | 3.47 (1.69 to 5.21) | 1.62 (1.38 to 1.86) |
| Central African Republic | 45 (21 to 77) | 4.42 (1.99 to 7.67) | 142 (66 to 254) | 7.12 (3.35 to 13.12) | 1.56 (1.51 to 1.62) |
| Chad | 77 (34 to 134) | 3.08 (1.33 to 5.4) | 217 (102 to 393) | 4.43 (2.04 to 7.86) | 1.01 (0.89 to 1.14) |
| Chile | 455 (251 to 653) | 5.06 (2.79 to 7.31) | 1956 (1030 to 2949) | 7.4 (3.89 to 11.14) | 1.61 (1.15 to 2.07) |
| China | 11598 (5254 to 22211) | 1.8 (0.82 to 3.42) | 54112 (27229 to 91108) | 2.83 (1.4 to 4.73) | 1.41 (1.31 to 1.51) |
| Colombia | 667 (331 to 1033) | 4.27 (2.1 to 6.7) | 2766 (1420 to 4337) | 4.91 (2.52 to 7.74) | 0.8 (0.63 to 0.97) |
| Comoros | 5 (2 to 10) | 3.22 (1.41 to 5.96) | 28 (13 to 52) | 6.64 (3.03 to 12.35) | 2.21 (2.1 to 2.31) |
| Congo | 63 (26 to 108) | 6.55 (2.7 to 11.55) | 245 (103 to 425) | 10.29 (4.23 to 17.92) | 1.21 (1.07 to 1.35) |
| Cook Islands | 1 (0 to 1) | 5.68 (2.56 to 9.56) | 2 (1 to 4) | 8.81 (4.28 to 14.12) | 1.47 (1.37 to 1.57) |
| Costa Rica | 73 (38 to 109) | 4.37 (2.29 to 6.57) | 553 (289 to 853) | 9.9 (5.22 to 15.24) | 2.77 (2.33 to 3.22) |
| Croatia | 150 (81 to 225) | 2.8 (1.51 to 4.19) | 490 (252 to 746) | 4.9 (2.52 to 7.45) | 1.85 (1.53 to 2.18) |
| Cuba | 210 (109 to 319) | 2.12 (1.11 to 3.21) | 1220 (687 to 1850) | 6.08 (3.44 to 9.14) | 4.17 (3.81 to 4.53) |
| Cyprus | 33 (15 to 59) | 7.33 (3.35 to 13.07) | 91 (42 to 160) | 5.96 (2.83 to 9.98) | -0.81 (-1.07 to -0.55) |
| Czechia | 380 (206 to 556) | 2.77 (1.53 to 4.07) | 578 (306 to 899) | 2.47 (1.3 to 3.82) | -0.29 (-0.49 to -0.08) |
| Côte d'Ivoire | 181 (84 to 303) | 5.59 (2.6 to 9.31) | 815 (400 to 1388) | 8.54 (4.04 to 14.11) | 1.22 (1.11 to 1.34) |
| Democratic People's Republic of Korea | 246 (104 to 451) | 2.01 (0.85 to 3.77) | 884 (397 to 1614) | 3.02 (1.36 to 5.5) | 1.45 (1.38 to 1.52) |
| Democratic Republic of the Congo | 708 (363 to 1197) | 5.66 (2.84 to 9.67) | 2880 (1367 to 5054) | 9.73 (4.65 to 17.05) | 1.59 (1.44 to 1.75) |
| Denmark | 96 (48 to 145) | 1.09 (0.55 to 1.64) | 506 (252 to 772) | 3.55 (1.77 to 5.41) | 3.94 (3.69 to 4.19) |
| Djibouti | 2 (1 to 4) | 1.77 (0.78 to 3.53) | 19 (9 to 37) | 3.95 (1.72 to 7.5) | 2.61 (2.5 to 2.71) |
| Dominica | 7 (4 to 10) | 12.11 (6.48 to 18.04) | 19 (11 to 28) | 23.98 (13.54 to 35.31) | 2.47 (2.38 to 2.56) |
| Dominican Republic | 160 (83 to 246) | 4.83 (2.46 to 7.46) | 939 (485 to 1441) | 9.48 (4.89 to 14.63) | 3.04 (2.77 to 3.31) |
| Ecuador | 375 (209 to 543) | 7.82 (4.28 to 11.58) | 2833 (1552 to 4440) | 18.39 (10.02 to 28.35) | 2.92 (2.02 to 3.82) |
| Egypt | 2922 (1591 to 4824) | 15.57 (8.31 to 26.13) | 14166 (7865 to 20836) | 30.74 (16.23 to 45.36) | 2.51 (2.41 to 2.62) |
| El Salvador | 207 (102 to 339) | 7.1 (3.47 to 11.74) | 1560 (828 to 2419) | 24.52 (12.94 to 38.09) | 4.09 (3.58 to 4.61) |
| Equatorial Guinea | 11 (5 to 19) | 6.5 (3.17 to 11.18) | 70 (30 to 125) | 16.47 (7.07 to 29.43) | 3.42 (3.02 to 3.83) |
| Eritrea | 12 (5 to 22) | 1.26 (0.53 to 2.41) | 60 (25 to 121) | 2.73 (1.12 to 5.43) | 2.61 (2.54 to 2.67) |
| Estonia | 25 (12 to 40) | 1.26 (0.62 to 2.03) | 182 (96 to 265) | 5.59 (2.93 to 8.22) | 4.54 (3.92 to 5.16) |
| Eswatini | 20 (10 to 33) | 8.88 (4.37 to 14.59) | 89 (44 to 147) | 19.6 (9.91 to 31.45) | 2.93 (2.25 to 3.61) |
| Ethiopia | 689 (306 to 1286) | 4.09 (1.84 to 7.58) | 1519 (673 to 2717) | 4.07 (1.76 to 7.4) | -0.43 (-0.6 to -0.26) |
| Fiji | 23 (11 to 40) | 7.54 (3.5 to 13.68) | 117 (54 to 187) | 19.11 (8.9 to 30.38) | 2.54 (2.12 to 2.96) |
| Finland | 60 (30 to 92) | 0.84 (0.42 to 1.28) | 246 (115 to 383) | 1.43 (0.68 to 2.23) | 2.24 (2.02 to 2.47) |
| France | 1704 (867 to 2665) | 1.9 (0.98 to 2.93) | 5069 (2497 to 7485) | 2.39 (1.18 to 3.52) | 1.24 (1 to 1.48) |
| Gabon | 51 (24 to 79) | 10.09 (4.76 to 15.82) | 196 (84 to 320) | 23.26 (9.28 to 39.49) | 2.63 (2.41 to 2.85) |
| Gambia | 15 (7 to 24) | 4.86 (2.21 to 8.12) | 77 (38 to 133) | 8.7 (4.22 to 15.49) | 1.69 (1.56 to 1.81) |
| Georgia | 42 (20 to 67) | 0.68 (0.33 to 1.09) | 176 (80 to 300) | 2.94 (1.33 to 4.93) | 5.04 (4.36 to 5.72) |
| Germany | 3010 (1447 to 4739) | 2.23 (1.08 to 3.5) | 11448 (4984 to 18852) | 4.41 (1.95 to 7.26) | 3.46 (2.98 to 3.94) |
| Ghana | 186 (85 to 317) | 3.69 (1.66 to 6.49) | 1522 (783 to 2530) | 11.43 (5.63 to 18.55) | 3.94 (3.85 to 4.03) |
| Greece | 831 (442 to 1235) | 5.88 (3.09 to 8.73) | 2430 (1223 to 3633) | 7.22 (3.7 to 10.67) | 0.09 (-0.89 to 1.08) |
| Greenland | 1 (0 to 1) | 4.1 (1.96 to 6.23) | 2 (1 to 4) | 4.75 (2.07 to 7.85) | 0.95 (0.76 to 1.15) |
| Grenada | 6 (3 to 10) | 8.38 (4.34 to 13.02) | 22 (12 to 34) | 19.8 (10.88 to 31.47) | 3.32 (3.11 to 3.53) |
| Guam | 4 (2 to 7) | 7.61 (3.88 to 11.75) | 18 (9 to 28) | 8.15 (4.08 to 12.87) | 1.1 (0.7 to 1.49) |
| Guatemala | 228 (117 to 336) | 8.36 (4.25 to 12.28) | 1634 (849 to 2621) | 15.6 (7.96 to 24.85) | 2.88 (2.46 to 3.3) |
| Guinea | 114 (54 to 197) | 3.84 (1.76 to 6.61) | 290 (131 to 510) | 5.68 (2.52 to 10.09) | 1.3 (1.24 to 1.36) |
| Guinea-Bissau | 20 (9 to 35) | 5.66 (2.67 to 9.98) | 48 (22 to 87) | 7.75 (3.44 to 13.74) | 0.9 (0.83 to 0.96) |
| Guyana | 31 (17 to 48) | 8.53 (4.76 to 13.54) | 130 (72 to 205) | 21.1 (11.53 to 33.24) | 4.09 (3.71 to 4.47) |
| Haiti | 93 (41 to 176) | 3.04 (1.32 to 5.88) | 372 (145 to 808) | 5.23 (2.04 to 11.64) | 2.13 (2 to 2.26) |
| Honduras | 52 (26 to 86) | 2.76 (1.34 to 4.58) | 415 (196 to 687) | 7.18 (3.4 to 11.89) | 3.39 (3.14 to 3.65) |
| Hungary | 256 (139 to 387) | 1.83 (1 to 2.79) | 732 (360 to 1153) | 3.39 (1.68 to 5.35) | 3.03 (2.71 to 3.36) |
| Iceland | 3 (2 to 5) | 1.09 (0.58 to 1.63) | 14 (7 to 22) | 2.01 (1 to 3.02) | 2.5 (2.31 to 2.7) |
| India | 3839 (1918 to 6552) | 0.91 (0.46 to 1.56) | 24249 (12246 to 39900) | 2.18 (1.09 to 3.62) | 2.85 (2.75 to 2.95) |
| Indonesia | 1250 (573 to 2175) | 1.33 (0.62 to 2.31) | 6952 (3105 to 11496) | 3 (1.37 to 4.96) | 2.82 (2.68 to 2.96) |
| Iran (Islamic Republic of) | 788 (392 to 1305) | 3.83 (1.85 to 6.55) | 4906 (2531 to 7439) | 7.23 (3.67 to 11.04) | 2.05 (1.91 to 2.18) |
| Iraq | 1022 (563 to 1562) | 13.6 (7.39 to 21.05) | 3284 (1590 to 5417) | 17.56 (8.41 to 28.74) | 0.44 (0.23 to 0.64) |
| Ireland | 80 (39 to 123) | 2.12 (1.04 to 3.21) | 235 (120 to 357) | 2.7 (1.38 to 4.1) | 1.48 (1.27 to 1.69) |
| Israel | 282 (143 to 426) | 6.47 (3.37 to 9.86) | 857 (403 to 1347) | 5.96 (2.78 to 9.39) | 0.36 (-0.14 to 0.86) |
| Italy | 1988 (1019 to 3029) | 2.28 (1.16 to 3.48) | 6243 (3047 to 9652) | 2.96 (1.44 to 4.55) | 0.81 (0.67 to 0.95) |
| Jamaica | 100 (47 to 163) | 5.47 (2.58 to 8.87) | 341 (171 to 536) | 10.61 (5.33 to 16.63) | 1.61 (0.97 to 2.25) |
| Japan | 3070 (1480 to 5016) | 2 (0.96 to 3.3) | 10715 (5087 to 17613) | 1.9 (0.92 to 3.09) | -0.26 (-0.38 to -0.14) |
| Jordan | 120 (64 to 182) | 11.24 (5.7 to 17.46) | 846 (495 to 1220) | 15.54 (8.68 to 22.56) | 0.88 (0.56 to 1.2) |
| Kazakhstan | 139 (69 to 224) | 1.07 (0.53 to 1.72) | 524 (254 to 873) | 3.23 (1.55 to 5.38) | 3.24 (2.86 to 3.62) |
| Kenya | 175 (81 to 317) | 2.47 (1.15 to 4.5) | 1259 (611 to 2071) | 6.62 (3.13 to 11.12) | 3.51 (3.41 to 3.61) |
| Kiribati | 2 (1 to 4) | 7.26 (3.17 to 12.67) | 9 (4 to 16) | 14.12 (5.66 to 27.2) | 2 (1.73 to 2.27) |
| Kuwait | 48 (27 to 72) | 9.7 (4.92 to 15.3) | 180 (102 to 261) | 8 (4.39 to 11.67) | -0.5 (-0.75 to -0.24) |
| Kyrgyzstan | 22 (11 to 37) | 0.71 (0.36 to 1.17) | 119 (62 to 187) | 2.52 (1.28 to 3.96) | 2.6 (1.67 to 3.53) |
| Lao People's Democratic Republic | 59 (27 to 108) | 3.13 (1.42 to 5.72) | 193 (75 to 368) | 4.52 (1.76 to 8.5) | 1.22 (1.19 to 1.24) |
| Latvia | 20 (9 to 31) | 0.57 (0.27 to 0.91) | 106 (55 to 163) | 2.4 (1.22 to 3.72) | 4.93 (4.45 to 5.42) |
| Lebanon | 171 (86 to 283) | 9.25 (4.61 to 15.2) | 860 (447 to 1339) | 13.1 (6.91 to 20.29) | 1.37 (1.2 to 1.55) |
| Lesotho | 22 (11 to 39) | 3.06 (1.39 to 5.33) | 109 (48 to 191) | 11.93 (5.13 to 20.86) | 5.55 (4.96 to 6.14) |
| Liberia | 83 (43 to 137) | 8 (4.01 to 12.98) | 240 (117 to 411) | 12.34 (5.89 to 21.59) | 1.67 (1.35 to 1.99) |
| Libya | 122 (59 to 204) | 7.02 (3.35 to 11.96) | 863 (407 to 1405) | 19.01 (8.85 to 30.93) | 4.04 (3.78 to 4.29) |
| Lithuania | 20 (10 to 32) | 0.45 (0.22 to 0.72) | 108 (58 to 162) | 1.73 (0.92 to 2.66) | 3.21 (2.72 to 3.7) |
| Luxembourg | 15 (8 to 22) | 2.9 (1.48 to 4.31) | 48 (21 to 79) | 3.75 (1.69 to 6.17) | 1.29 (1.01 to 1.58) |
| Madagascar | 75 (33 to 141) | 1.78 (0.78 to 3.39) | 282 (116 to 513) | 3.34 (1.35 to 5.97) | 2.04 (1.91 to 2.17) |
| Malawi | 107 (46 to 199) | 3.42 (1.5 to 6.49) | 413 (196 to 720) | 6.69 (3.08 to 11.8) | 2.02 (1.81 to 2.23) |
| Malaysia | 378 (197 to 603) | 4.25 (2.24 to 6.76) | 2089 (1095 to 3213) | 7.91 (4.11 to 12.19) | 1.78 (1.58 to 1.99) |
| Maldives | 5 (2 to 8) | 5.27 (2.32 to 9.67) | 15 (7 to 26) | 4.56 (1.99 to 8.11) | -0.79 (-1.01 to -0.57) |
| Mali | 165 (81 to 283) | 4.95 (2.41 to 8.39) | 449 (232 to 768) | 5.98 (2.9 to 10.31) | 0.68 (0.52 to 0.83) |
| Malta | 10 (5 to 16) | 2.77 (1.39 to 4.28) | 41 (19 to 67) | 3.51 (1.62 to 5.73) | 0.86 (0.54 to 1.19) |
| Marshall Islands | 1 (0 to 2) | 7.55 (2.93 to 15.93) | 5 (1 to 12) | 15.3 (3.45 to 43.5) | 2.24 (2.01 to 2.47) |
| Mauritania | 81 (40 to 131) | 9.64 (4.6 to 15.54) | 241 (118 to 402) | 13.58 (6.63 to 22.67) | 0.75 (0.58 to 0.93) |
| Mauritius | 50 (24 to 81) | 7.22 (3.42 to 11.97) | 380 (189 to 607) | 20.91 (10.29 to 33.26) | 3.7 (3.27 to 4.14) |
| Mexico | 3595 (1870 to 5598) | 9.92 (5.14 to 15.89) | 24462 (13956 to 35479) | 19.85 (11.27 to 28.91) | 2.84 (2.15 to 3.53) |
| Micronesia (Federated States of) | 4 (2 to 7) | 8.81 (3.92 to 17.19) | 12 (5 to 21) | 18 (7.09 to 32.95) | 2.32 (1.93 to 2.7) |
| Monaco | 2 (1 to 3) | 1.95 (1.03 to 2.98) | 5 (2 to 7) | 3.51 (1.81 to 5.23) | 2.11 (1.83 to 2.4) |
| Mongolia | 31 (16 to 50) | 3.03 (1.55 to 4.87) | 74 (40 to 122) | 3.46 (1.85 to 5.82) | 0.02 (-0.13 to 0.18) |
| Montenegro | 27 (15 to 40) | 4.54 (2.48 to 6.85) | 67 (36 to 102) | 7.68 (3.93 to 11.63) | 1.88 (1.6 to 2.16) |
| Morocco | 772 (374 to 1428) | 5.98 (2.9 to 11.32) | 3845 (1984 to 6260) | 12.64 (6.47 to 20.62) | 2.7 (2.58 to 2.83) |
| Mozambique | 83 (34 to 150) | 1.68 (0.7 to 3.16) | 374 (162 to 699) | 3.94 (1.69 to 7.4) | 3.22 (3.08 to 3.36) |
| Myanmar | 485 (202 to 854) | 2.17 (0.94 to 3.81) | 1315 (596 to 2336) | 2.82 (1.28 to 4.93) | 0.64 (0.57 to 0.72) |
| Namibia | 19 (9 to 33) | 3.59 (1.63 to 6.16) | 89 (45 to 147) | 7.99 (4.06 to 13.08) | 2.42 (2.07 to 2.77) |
| Nauru | 0 (0 to 1) | 10.6 (4.69 to 19.47) | 1 (1 to 2) | 22.05 (9.54 to 39.94) | 2.21 (2.08 to 2.33) |
| Nepal | 85 (38 to 150) | 0.93 (0.4 to 1.66) | 498 (234 to 905) | 2.2 (1.07 to 4.01) | 3.13 (2.8 to 3.46) |
| Netherlands | 331 (158 to 519) | 1.63 (0.78 to 2.56) | 1386 (701 to 2138) | 3.36 (1.7 to 5.17) | 2.58 (2.2 to 2.96) |
| New Zealand | 84 (45 to 126) | 2.24 (1.2 to 3.33) | 365 (201 to 525) | 3.99 (2.22 to 5.72) | 2.16 (1.71 to 2.6) |
| Nicaragua | 104 (49 to 163) | 7.14 (3.34 to 11.48) | 849 (429 to 1375) | 17.9 (8.96 to 29.02) | 3.6 (3.17 to 4.03) |
| Niger | 69 (32 to 121) | 2.97 (1.36 to 5.15) | 230 (102 to 417) | 3.32 (1.47 to 5.94) | 0.26 (0.15 to 0.37) |
| Nigeria | 1503 (736 to 2520) | 4.17 (1.99 to 7.01) | 5378 (2703 to 8724) | 7.53 (3.72 to 12.05) | 1.75 (1.66 to 1.84) |
| Niue | 0 (0 to 0) | 8.16 (3.64 to 14.52) | 0 (0 to 1) | 20.51 (7.77 to 37.98) | 2.99 (2.79 to 3.19) |
| North Macedonia | 49 (26 to 78) | 2.88 (1.5 to 4.52) | 126 (61 to 208) | 4.54 (2.26 to 7.44) | 1.46 (1.11 to 1.82) |
| Northern Mariana Islands | 2 (1 to 3) | 12.34 (5.45 to 21.09) | 9 (4 to 15) | 22.13 (9.62 to 36.87) | 2.06 (1.87 to 2.25) |
| Norway | 71 (37 to 110) | 0.92 (0.47 to 1.42) | 243 (125 to 379) | 1.93 (0.99 to 3) | 2.72 (2.38 to 3.06) |
| Oman | 29 (13 to 48) | 4.9 (2.16 to 8.41) | 259 (146 to 392) | 17.43 (9.75 to 26.44) | 5 (4.65 to 5.35) |
| Pakistan | 1307 (646 to 2181) | 2.5 (1.23 to 4.21) | 6252 (2948 to 10489) | 5.47 (2.62 to 9.11) | 2.5 (2.24 to 2.76) |
| Palau | 1 (0 to 1) | 9.12 (4.26 to 15.96) | 3 (2 to 6) | 19 (8.6 to 32.33) | 2.57 (2.36 to 2.78) |
| Palestine | 98 (50 to 166) | 13.46 (6.81 to 23.07) | 320 (172 to 486) | 16.99 (8.7 to 26.08) | 0.7 (0.46 to 0.93) |
| Panama | 59 (31 to 86) | 4.26 (2.24 to 6.19) | 532 (284 to 791) | 11.79 (6.28 to 17.56) | 3.62 (3.15 to 4.09) |
| Papua New Guinea | 24 (10 to 49) | 1.41 (0.57 to 2.8) | 88 (35 to 176) | 1.77 (0.7 to 3.64) | 0.64 (0.57 to 0.72) |
| Paraguay | 123 (66 to 181) | 5.92 (3.19 to 8.87) | 587 (296 to 909) | 10.61 (5.3 to 16.49) | 2.18 (2.05 to 2.31) |
| Peru | 868 (443 to 1288) | 7.82 (3.96 to 11.68) | 3987 (2045 to 6223) | 11.94 (6.09 to 18.68) | 1.13 (0.92 to 1.34) |
| Philippines | 674 (300 to 1161) | 2.68 (1.19 to 4.61) | 4725 (1969 to 8254) | 6.03 (2.51 to 10.56) | 3.11 (2.92 to 3.31) |
| Poland | 1550 (812 to 2307) | 3.7 (1.94 to 5.53) | 1699 (933 to 2649) | 2.21 (1.21 to 3.45) | -1.89 (-2.5 to -1.27) |
| Portugal | 420 (219 to 659) | 3.38 (1.76 to 5.32) | 1526 (742 to 2398) | 4.59 (2.26 to 7.2) | 0.96 (0.46 to 1.45) |
| Puerto Rico | 402 (226 to 608) | 11.81 (6.68 to 17.95) | 1187 (678 to 1821) | 14.66 (8.67 to 21.86) | 1.41 (0.99 to 1.83) |
| Qatar | 8 (4 to 15) | 12.66 (5.48 to 23.42) | 92 (57 to 134) | 19.81 (11.6 to 28.62) | 1.01 (0.35 to 1.68) |
| Republic of Korea | 527 (235 to 885) | 2.53 (1.09 to 4.3) | 2063 (953 to 3472) | 2.27 (1.05 to 3.83) | -0.18 (-0.36 to 0.01) |
| Republic of Moldova | 16 (8 to 25) | 0.4 (0.2 to 0.63) | 66 (36 to 103) | 1.11 (0.59 to 1.73) | 2.78 (2.3 to 3.27) |
| Romania | 732 (394 to 1142) | 2.92 (1.54 to 4.54) | 1258 (725 to 1851) | 3.2 (1.84 to 4.7) | 0.63 (0.15 to 1.12) |
| Russian Federation | 1644 (852 to 2558) | 0.94 (0.48 to 1.44) | 4603 (2479 to 6740) | 1.9 (1.02 to 2.79) | 1.92 (1.45 to 2.4) |
| Rwanda | 72 (30 to 136) | 2.95 (1.27 to 5.57) | 196 (79 to 372) | 3.84 (1.56 to 7.34) | 0 (-0.37 to 0.38) |
| Saint Kitts and Nevis | 4 (2 to 7) | 12.52 (6.62 to 19.99) | 14 (8 to 21) | 23.07 (12.56 to 35.02) | 2.94 (2.63 to 3.25) |
| Saint Lucia | 7 (4 to 11) | 8.74 (4.39 to 13.91) | 32 (17 to 50) | 13.66 (7.09 to 21.53) | 1.76 (1.47 to 2.06) |
| Saint Vincent and the Grenadines | 4 (2 to 6) | 5.42 (2.67 to 8.73) | 17 (9 to 27) | 12.52 (6.46 to 19.95) | 3.45 (3.1 to 3.79) |
| Samoa | 7 (3 to 12) | 8.97 (3.9 to 16.18) | 22 (10 to 35) | 16.56 (7.46 to 26.91) | 1.99 (1.9 to 2.07) |
| San Marino | 1 (0 to 1) | 1.66 (0.85 to 2.55) | 2 (1 to 3) | 1.35 (0.61 to 2.27) | 0.62 (0.18 to 1.05) |
| Sao Tome and Principe | 5 (3 to 9) | 9.29 (4.55 to 14.99) | 18 (9 to 29) | 19.66 (9.16 to 31.44) | 2.46 (2.4 to 2.52) |
| Saudi Arabia | 667 (338 to 1108) | 12.89 (6.27 to 21.61) | 5336 (3151 to 7851) | 35.62 (19.8 to 53.06) | 3.03 (2.82 to 3.25) |
| Senegal | 182 (90 to 298) | 6.45 (3.24 to 10.58) | 593 (281 to 1003) | 8.88 (4.17 to 14.85) | 0.83 (0.71 to 0.96) |
| Serbia | 419 (221 to 679) | 4.65 (2.43 to 7.67) | 996 (531 to 1546) | 5.73 (3.06 to 8.91) | 0.63 (0.5 to 0.76) |
| Seychelles | 3 (2 to 6) | 6.21 (3.18 to 9.88) | 16 (8 to 25) | 14.63 (6.9 to 23.83) | 2.97 (2.73 to 3.22) |
| Sierra Leone | 67 (33 to 115) | 3.72 (1.85 to 6.43) | 163 (78 to 285) | 4.97 (2.39 to 8.57) | 0.88 (0.84 to 0.91) |
| Singapore | 35 (17 to 57) | 1.83 (0.87 to 3.08) | 184 (84 to 304) | 2.22 (1.01 to 3.68) | 2.04 (1.46 to 2.62) |
| Slovakia | 210 (117 to 316) | 3.56 (1.98 to 5.36) | 330 (183 to 489) | 3.43 (1.91 to 5.09) | -0.04 (-0.1 to 0.01) |
| Slovenia | 47 (25 to 72) | 1.95 (1.03 to 2.99) | 128 (68 to 197) | 2.35 (1.25 to 3.66) | 1.41 (1.1 to 1.71) |
| Solomon Islands | 5 (2 to 10) | 3.73 (1.31 to 8.14) | 14 (5 to 28) | 4.24 (1.5 to 8.85) | 0.16 (-0.04 to 0.36) |
| Somalia | 63 (29 to 124) | 3.1 (1.39 to 6.04) | 258 (112 to 488) | 4.89 (2.07 to 9.3) | 1.61 (1.52 to 1.69) |
| South Africa | 822 (409 to 1334) | 4.28 (2.12 to 7.05) | 4571 (2380 to 6980) | 11.38 (5.84 to 17.64) | 3.07 (2.61 to 3.53) |
| South Sudan | 52 (23 to 110) | 2.34 (1.02 to 4.83) | 147 (61 to 285) | 4.55 (1.87 to 8.67) | 2.1 (1.88 to 2.32) |
| Spain | 1913 (948 to 3013) | 3.65 (1.82 to 5.73) | 4891 (2225 to 7850) | 3.4 (1.59 to 5.42) | -0.04 (-0.14 to 0.06) |
| Sri Lanka | 231 (100 to 412) | 2.44 (1.02 to 4.36) | 895 (359 to 1740) | 3.53 (1.4 to 6.83) | 1.19 (1 to 1.38) |
| Sudan | 408 (200 to 738) | 4.87 (2.33 to 9.08) | 1802 (856 to 3048) | 10.5 (4.92 to 17.56) | 2.58 (2.37 to 2.79) |
| Suriname | 16 (9 to 26) | 6.41 (3.33 to 10.4) | 79 (39 to 132) | 12.63 (6.21 to 21.3) | 2.55 (2.35 to 2.75) |
| Sweden | 157 (78 to 242) | 0.91 (0.45 to 1.42) | 733 (362 to 1177) | 2.51 (1.25 to 4.02) | 3.72 (3.55 to 3.88) |
| Switzerland | 201 (92 to 322) | 1.76 (0.82 to 2.8) | 649 (274 to 1116) | 2.49 (1.06 to 4.3) | 2.15 (1.8 to 2.5) |
| Syrian Arab Republic | 513 (274 to 830) | 11.38 (5.94 to 18.36) | 2104 (1086 to 3298) | 20.37 (10.29 to 31.8) | 1.51 (1.25 to 1.77) |
| Taiwan (Province of China) | 611 (276 to 1052) | 5.21 (2.38 to 9.17) | 2537 (1163 to 4423) | 5.61 (2.57 to 9.76) | 0.58 (0.26 to 0.9) |
| Tajikistan | 9 (4 to 16) | 0.33 (0.16 to 0.57) | 33 (15 to 57) | 0.58 (0.26 to 0.98) | 1.34 (0.92 to 1.77) |
| Thailand | 746 (325 to 1324) | 2.37 (1.04 to 4.17) | 6240 (2547 to 11055) | 5.71 (2.32 to 10.13) | 2.54 (2.32 to 2.76) |
| Timor-Leste | 3 (1 to 6) | 1.21 (0.48 to 2.38) | 15 (6 to 30) | 1.9 (0.79 to 3.84) | 1.76 (1.42 to 2.1) |
| Togo | 43 (21 to 72) | 4.32 (2.04 to 7.25) | 220 (106 to 376) | 7.62 (3.64 to 13.23) | 1.68 (1.61 to 1.75) |
| Tokelau | 0 (0 to 0) | 5.88 (2.68 to 11.47) | 0 (0 to 0) | 11.91 (5.17 to 20.71) | 2.35 (2.18 to 2.53) |
| Tonga | 2 (1 to 4) | 4.09 (1.67 to 7.34) | 7 (3 to 11) | 8.43 (3.52 to 13.95) | 2.24 (2.01 to 2.46) |
| Trinidad and Tobago | 53 (28 to 80) | 6.9 (3.7 to 10.56) | 304 (160 to 481) | 15.82 (8.33 to 25.07) | 3.39 (3.02 to 3.77) |
| Tunisia | 172 (82 to 296) | 4.02 (1.9 to 6.99) | 1203 (578 to 2042) | 10.12 (4.91 to 17.52) | 3.1 (3.03 to 3.16) |
| Turkmenistan | 36 (19 to 57) | 1.78 (0.95 to 2.83) | 192 (97 to 330) | 4.76 (2.36 to 8.18) | 2.85 (2.27 to 3.43) |
| Tuvalu | 0 (0 to 1) | 5.13 (1.98 to 10.48) | 1 (0 to 2) | 11.1 (4.14 to 22.82) | 2.58 (2.47 to 2.68) |
| Türkiye | 2731 (1438 to 4313) | 9.42 (4.9 to 14.83) | 10281 (5592 to 15680) | 12.11 (6.54 to 18.48) | 1.24 (0.78 to 1.71) |
| Uganda | 127 (54 to 237) | 2.26 (0.99 to 4.18) | 558 (245 to 1034) | 4.39 (1.98 to 8.3) | 1.81 (1.64 to 1.98) |
| Ukraine | 16 (8 to 25) | 0.02 (0.01 to 0.04) | 409 (200 to 684) | 0.56 (0.27 to 0.96) | 14.26 (12.24 to 16.31) |
| United Arab Emirates | 23 (12 to 39) | 6.29 (3.06 to 11.17) | 313 (169 to 449) | 17.94 (8.32 to 26.52) | 5.92 (5.12 to 6.73) |
| United Kingdom | 998 (450 to 1521) | 1.05 (0.47 to 1.6) | 2486 (1093 to 3997) | 1.55 (0.68 to 2.47) | 2.07 (1.75 to 2.39) |
| United Republic of Tanzania | 361 (177 to 620) | 3.94 (1.83 to 6.67) | 1637 (835 to 2785) | 7.45 (3.79 to 12.47) | 2.04 (1.99 to 2.09) |
| United States of America | 10066 (5407 to 14042) | 3.04 (1.65 to 4.23) | 62324 (33545 to 89159) | 9.98 (5.47 to 14.15) | 4.27 (4.04 to 4.49) |
| United States Virgin Islands | 6 (3 to 9) | 7.9 (4.38 to 11.84) | 16 (8 to 25) | 9.44 (4.93 to 14.49) | 0.94 (0.66 to 1.21) |
| Uruguay | 145 (70 to 218) | 3.73 (1.81 to 5.58) | 353 (172 to 553) | 5.37 (2.62 to 8.34) | 1.51 (1.25 to 1.76) |
| Uzbekistan | 111 (47 to 219) | 1.04 (0.43 to 2.06) | 901 (441 to 1505) | 3.61 (1.76 to 6.08) | 3 (2.16 to 3.85) |
| Vanuatu | 3 (1 to 5) | 5.18 (2.33 to 10.16) | 13 (5 to 24) | 8.17 (3.42 to 15.58) | 1.41 (1.35 to 1.46) |
| Venezuela (Bolivarian Republic of) | 447 (240 to 663) | 4.97 (2.64 to 7.36) | 4232 (2202 to 6691) | 14.5 (7.49 to 23.06) | 3.19 (2.74 to 3.65) |
| Viet Nam | 384 (163 to 716) | 1.05 (0.44 to 1.96) | 1943 (789 to 3588) | 2.18 (0.86 to 3.99) | 2.76 (2.56 to 2.95) |
| Yemen | 124 (59 to 240) | 2.97 (1.45 to 5.79) | 618 (297 to 1117) | 5.31 (2.52 to 9.8) | 1.79 (1.56 to 2.03) |
| Zambia | 102 (47 to 181) | 4.17 (1.95 to 7.58) | 535 (253 to 971) | 8.77 (4.06 to 15.82) | 2.06 (1.87 to 2.25) |
| Zimbabwe | 97 (43 to 175) | 2.89 (1.25 to 5.2) | 521 (243 to 898) | 8.87 (4.04 to 15.45) | 4.19 (3.61 to 4.78) |

Abbreviations: BMI, body mass index; ASMR, age-standardized mortality rate; EAPC, estimated annual percentage change.

**Supplementary Table 3. DALYs of chronic kidney disease** **attributable to high BMI in 1990 and 2021, and the estimated annual percentage changes from 1990 to 2021.**

| **Location** | **Number, 1990** | **ASDR, 1990** | **Number, 2021** | **ASDR, 2021** | **EAPC, 1990-2021** |
| --- | --- | --- | --- | --- | --- |
| Afghanistan | 18210 (9208 to 30928) | 251.76 (127.75 to 426.85) | 38005 (16015 to 70229) | 349.2 (147.42 to 672.03) | 1.15 (0.99 to 1.31) |
| Albania | 1773 (987 to 2737) | 88.54 (49.8 to 135.77) | 3948 (2218 to 5875) | 95.01 (53.11 to 142.54) | 0.44 (0.25 to 0.62) |
| Algeria | 14503 (7405 to 26270) | 129.1 (64.66 to 236.71) | 80765 (44360 to 121727) | 245.26 (133.86 to 367.67) | 2.32 (2.12 to 2.52) |
| American Samoa | 62 (30 to 101) | 264.65 (120.49 to 442.23) | 321 (156 to 487) | 668.9 (321.39 to 1029.32) | 3.19 (2.95 to 3.43) |
| Andorra | 38 (20 to 62) | 73.29 (38.02 to 116.98) | 110 (57 to 174) | 67.42 (35.34 to 106.49) | -0.03 (-0.2 to 0.13) |
| Angola | 3450 (1490 to 6378) | 80.61 (35.1 to 149.52) | 18177 (7999 to 32143) | 138.36 (60.69 to 246.08) | 1.55 (1.4 to 1.7) |
| Antigua and Barbuda | 110 (59 to 170) | 212.74 (115.51 to 327.2) | 429 (248 to 624) | 399.67 (228.97 to 585.87) | 2.67 (2.42 to 2.93) |
| Argentina | 60441 (33276 to 85796) | 190.45 (103.96 to 270.98) | 117059 (66099 to 165983) | 208.13 (117.73 to 296.09) | 0.53 (0.21 to 0.84) |
| Armenia | 1157 (613 to 1806) | 42.36 (22.53 to 66.48) | 4565 (2417 to 6939) | 107.26 (56.73 to 162.38) | 3.25 (2.92 to 3.59) |
| Australia | 10062 (5229 to 15310) | 52.94 (27.58 to 80.7) | 35023 (19627 to 49395) | 73.27 (41.61 to 102.96) | 1.45 (1.26 to 1.64) |
| Austria | 5624 (2868 to 8396) | 47.52 (24.39 to 70.36) | 18163 (9611 to 26300) | 86.8 (46.08 to 125.73) | 2.99 (2.59 to 3.39) |
| Azerbaijan | 4121 (2252 to 6420) | 78.37 (42.83 to 120.74) | 13588 (7707 to 21130) | 125.4 (68.67 to 197.58) | 1.67 (1.52 to 1.83) |
| Bahamas | 385 (218 to 531) | 227.91 (126.54 to 320.32) | 1717 (1000 to 2413) | 404.76 (233.52 to 573.79) | 2.44 (2.24 to 2.64) |
| Bahrain | 398 (214 to 671) | 225.69 (117.44 to 392.95) | 3185 (1944 to 4494) | 410.01 (243.39 to 604.21) | 1.53 (1.23 to 1.83) |
| Bangladesh | 12073 (5066 to 22298) | 23.58 (10.13 to 43.47) | 60310 (29134 to 102733) | 42.1 (20.31 to 72.26) | 2.39 (2.21 to 2.58) |
| Barbados | 493 (282 to 718) | 182.21 (105.78 to 261.38) | 1509 (862 to 2261) | 304.95 (176.39 to 449.3) | 2.18 (1.92 to 2.44) |
| Belarus | 2980 (1446 to 4725) | 23.7 (11.47 to 37.65) | 6526 (3601 to 9780) | 42.01 (23.07 to 63.26) | 1.87 (1.59 to 2.14) |
| Belgium | 8731 (4419 to 12950) | 56.66 (28.61 to 83.56) | 17902 (9002 to 26260) | 69.91 (36.15 to 102.23) | 1.17 (0.98 to 1.37) |
| Belize | 211 (119 to 289) | 219.33 (123.04 to 302.31) | 1533 (954 to 2075) | 476.82 (287.2 to 657.71) | 2.95 (2.51 to 3.4) |
| Benin | 2257 (1010 to 3729) | 109.89 (49.14 to 181.75) | 9691 (4473 to 16179) | 170.52 (78.86 to 282.52) | 1.33 (1.23 to 1.43) |
| Bermuda | 105 (62 to 140) | 167.93 (98.43 to 224.35) | 244 (147 to 334) | 184.56 (115.27 to 250.49) | 0.76 (0.49 to 1.03) |
| Bhutan | 237 (112 to 397) | 85.62 (41.05 to 145.26) | 719 (333 to 1221) | 113.04 (52.26 to 192.93) | 0.91 (0.84 to 0.97) |
| Bolivia (Plurinational State of) | 7959 (3841 to 12420) | 243.72 (118.81 to 380.32) | 38771 (21299 to 59926) | 424.28 (229.4 to 660.3) | 1.88 (1.79 to 1.97) |
| Bosnia and Herzegovina | 2901 (1536 to 4450) | 71.3 (37.87 to 109.41) | 5372 (2684 to 8384) | 88.23 (43.93 to 138.4) | 0.66 (0.49 to 0.83) |
| Botswana | 432 (198 to 755) | 77.27 (35.17 to 136.74) | 2667 (1279 to 4461) | 180.13 (85.66 to 294.21) | 3.04 (2.8 to 3.27) |
| Brazil | 137857 (76545 to 196760) | 148.6 (81.2 to 215.29) | 494253 (286017 to 670611) | 196.58 (113.18 to 268.03) | 0.8 (0.61 to 1) |
| Brunei Darussalam | 110 (52 to 186) | 100.29 (46.71 to 169.18) | 522 (269 to 818) | 144.52 (72.52 to 227.23) | 1.53 (1.39 to 1.66) |
| Bulgaria | 8594 (4768 to 12350) | 76.26 (42.28 to 110.85) | 21023 (11483 to 31991) | 159.73 (86.52 to 242.89) | 2.97 (2.63 to 3.3) |
| Burkina Faso | 2882 (1230 to 5118) | 65.43 (28.47 to 116.87) | 9420 (4167 to 16779) | 94.72 (40.68 to 167.64) | 1.24 (1.15 to 1.33) |
| Burundi | 1225 (552 to 2273) | 51.92 (23.67 to 95.7) | 3120 (1456 to 5920) | 62.8 (28.55 to 116.86) | 0.13 (-0.05 to 0.32) |
| Cabo Verde | 144 (68 to 235) | 64.56 (30.5 to 103.94) | 755 (366 to 1268) | 159.88 (76.17 to 271.18) | 2.78 (2.62 to 2.93) |
| Cambodia | 1664 (702 to 3020) | 34.62 (14.65 to 63.78) | 6209 (2485 to 11700) | 47.65 (19.34 to 87.1) | 0.93 (0.71 to 1.14) |
| Cameroon | 12125 (5951 to 19142) | 271.05 (128.38 to 428.96) | 55674 (28263 to 93590) | 414.35 (210.71 to 690.39) | 0.82 (0.54 to 1.09) |
| Canada | 16452 (8519 to 23514) | 50.96 (26.33 to 72.64) | 51604 (25800 to 74930) | 70.55 (35.91 to 100.38) | 1.39 (1.19 to 1.59) |
| Central African Republic | 1531 (691 to 2599) | 119.65 (54.13 to 203.86) | 5087 (2341 to 8963) | 191.89 (90.39 to 337.19) | 1.53 (1.47 to 1.58) |
| Chad | 2096 (942 to 3520) | 75.23 (34.4 to 125.68) | 6565 (3077 to 11565) | 107.44 (50.51 to 190.4) | 0.98 (0.84 to 1.11) |
| Chile | 11600 (6531 to 16765) | 117.29 (66.11 to 169.33) | 37523 (20610 to 54668) | 145.38 (80.1 to 211.69) | 1.01 (0.59 to 1.42) |
| China | 349224 (157171 to 671163) | 43.49 (19.71 to 82.98) | 1385819 (702691 to 2257375) | 66.97 (33.82 to 109.54) | 1.44 (1.32 to 1.56) |
| Colombia | 19854 (9925 to 30494) | 110.58 (54.86 to 168.99) | 69548 (35646 to 103957) | 124.73 (63.95 to 186.93) | 0.62 (0.46 to 0.77) |
| Comoros | 149 (65 to 268) | 74.13 (32.65 to 132.91) | 722 (315 to 1307) | 146.4 (64.44 to 264.86) | 2.03 (1.91 to 2.16) |
| Congo | 1976 (812 to 3331) | 172.35 (70.86 to 287.81) | 8052 (3613 to 14081) | 259.31 (113.65 to 444.17) | 1.05 (0.91 to 1.19) |
| Cook Islands | 19 (9 to 30) | 151.97 (73.18 to 242.19) | 55 (28 to 85) | 217.94 (108.55 to 330.34) | 1.25 (1.17 to 1.32) |
| Costa Rica | 2267 (1192 to 3385) | 126.58 (67.07 to 190.02) | 14039 (7724 to 20761) | 253.04 (139.15 to 375.16) | 2.3 (1.94 to 2.66) |
| Côte d'Ivoire | 5995 (2792 to 10102) | 135.93 (64.09 to 224.66) | 26492 (12715 to 45922) | 207.15 (102.01 to 351.76) | 1.21 (1.09 to 1.32) |
| Croatia | 4459 (2468 to 6620) | 76.76 (42.46 to 114.72) | 9763 (5117 to 14647) | 107.47 (56.65 to 161.41) | 1.06 (0.85 to 1.27) |
| Cuba | 6800 (3604 to 9962) | 66.5 (35.28 to 97.73) | 29264 (17039 to 42193) | 155.66 (91.5 to 221.5) | 3.32 (3.02 to 3.62) |
| Cyprus | 682 (322 to 1153) | 114.52 (54 to 194.28) | 1802 (856 to 2983) | 100.46 (48.58 to 164.7) | -0.53 (-0.7 to -0.36) |
| Czechia | 11493 (6519 to 16515) | 85.39 (48.65 to 122.86) | 14393 (7795 to 21727) | 67.7 (37.33 to 101.92) | -0.67 (-0.79 to -0.54) |
| Democratic People's Republic of Korea | 7033 (2963 to 12872) | 47.51 (20.13 to 86.95) | 22043 (9677 to 40675) | 69.85 (30.66 to 128.2) | 1.31 (1.27 to 1.34) |
| Democratic Republic of the Congo | 22033 (10993 to 36930) | 137.88 (70.46 to 231.91) | 88969 (42912 to 152590) | 230.88 (110.99 to 394.07) | 1.5 (1.36 to 1.63) |
| Denmark | 3097 (1529 to 4637) | 38.03 (18.74 to 57.25) | 9300 (4799 to 13951) | 73.4 (38.53 to 109.8) | 2.08 (1.94 to 2.22) |
| Djibouti | 56 (25 to 111) | 40.01 (17.92 to 77.64) | 574 (251 to 1076) | 86.78 (38.46 to 162.95) | 2.5 (2.38 to 2.61) |
| Dominica | 164 (89 to 237) | 284.1 (154.75 to 409.1) | 457 (264 to 657) | 550.83 (316.24 to 794.71) | 2.42 (2.33 to 2.52) |
| Dominican Republic | 4845 (2563 to 7284) | 124.13 (64.41 to 186.04) | 25856 (13827 to 38106) | 251.19 (134.15 to 369.54) | 3.04 (2.83 to 3.25) |
| Ecuador | 9579 (5489 to 13380) | 178.27 (100.92 to 251.85) | 64179 (35786 to 102752) | 395.55 (219.91 to 629.74) | 2.61 (1.76 to 3.46) |
| Egypt | 73219 (41529 to 118068) | 303.26 (167.06 to 488.17) | 358342 (202168 to 518336) | 611.76 (335.91 to 896.36) | 2.52 (2.42 to 2.62) |
| El Salvador | 5896 (3071 to 9210) | 192.13 (99.47 to 301.88) | 38248 (21021 to 58232) | 622.23 (343.55 to 948.55) | 3.97 (3.5 to 4.44) |
| Equatorial Guinea | 355 (170 to 597) | 172.24 (84.08 to 287.29) | 2197 (1013 to 3789) | 375.16 (167.45 to 655.37) | 2.83 (2.47 to 3.19) |
| Eritrea | 383 (156 to 746) | 31.35 (12.67 to 59.25) | 1819 (723 to 3650) | 62.77 (25.9 to 124.18) | 2.35 (2.29 to 2.4) |
| Estonia | 1158 (570 to 1807) | 58.56 (28.73 to 91.69) | 3746 (2025 to 5393) | 133.97 (71.79 to 193.92) | 2.44 (2.04 to 2.84) |
| Eswatini | 572 (294 to 903) | 203.7 (102.38 to 327.39) | 2673 (1292 to 4509) | 462.92 (227.14 to 753.86) | 2.95 (2.26 to 3.64) |
| Ethiopia | 18977 (8123 to 35408) | 94.13 (41.27 to 174.49) | 38209 (16973 to 67476) | 88.07 (39.36 to 156.66) | -0.7 (-0.88 to -0.51) |
| Fiji | 735 (358 to 1260) | 194.77 (94.76 to 333.66) | 3199 (1491 to 4956) | 427.12 (200.31 to 667.41) | 2.19 (1.88 to 2.51) |
| Finland | 2282 (1206 to 3476) | 32.42 (17.04 to 49.55) | 5417 (2706 to 8378) | 39.78 (19.59 to 61.12) | 1.06 (0.9 to 1.22) |
| France | 34471 (17383 to 52624) | 40.34 (20.28 to 61.63) | 84765 (43269 to 124178) | 50.95 (26.24 to 74.32) | 0.86 (0.76 to 0.97) |
| Gabon | 1426 (697 to 2254) | 248.49 (119.97 to 389.26) | 5498 (2517 to 9049) | 513.58 (229.09 to 838.96) | 2.26 (2.03 to 2.49) |
| Gambia | 445 (216 to 718) | 119.41 (56.64 to 194.29) | 2272 (1120 to 3851) | 211.04 (104.21 to 358.34) | 1.62 (1.47 to 1.77) |
| Georgia | 3708 (1944 to 5775) | 60.67 (31.84 to 94.43) | 6471 (3233 to 10427) | 115.3 (57.62 to 185.77) | 2.4 (2.05 to 2.75) |
| Germany | 79521 (39058 to 122566) | 62.6 (30.7 to 96.22) | 180221 (83148 to 289597) | 81.21 (38.95 to 127.16) | 1.39 (1.18 to 1.6) |
| Ghana | 5377 (2476 to 9151) | 83.91 (38.03 to 140.66) | 41357 (21244 to 67620) | 242.44 (125.47 to 398.04) | 3.72 (3.63 to 3.81) |
| Greece | 16240 (8644 to 23775) | 109.8 (57.92 to 160) | 38071 (20004 to 55684) | 136.71 (72.69 to 199.2) | 0.43 (-0.19 to 1.05) |
| Greenland | 28 (14 to 42) | 90.37 (43.61 to 135.78) | 65 (31 to 103) | 100.9 (46.82 to 162.09) | 0.77 (0.61 to 0.92) |
| Grenada | 155 (81 to 237) | 234.06 (124.95 to 351.78) | 576 (331 to 875) | 493.77 (282.03 to 749.08) | 3.02 (2.82 to 3.22) |
| Guam | 131 (69 to 197) | 175.32 (90.51 to 265.14) | 511 (261 to 790) | 243.92 (126.48 to 372.96) | 1.75 (1.47 to 2.03) |
| Guatemala | 6686 (3506 to 9959) | 193.08 (100.58 to 283.82) | 46518 (24254 to 72573) | 405.21 (211.09 to 636.48) | 3.29 (2.84 to 3.74) |
| Guinea | 3265 (1551 to 5565) | 97.21 (45.81 to 166.26) | 8797 (4057 to 15617) | 143.11 (65.11 to 248.63) | 1.25 (1.2 to 1.31) |
| Guinea-Bissau | 607 (278 to 1072) | 143.55 (66.48 to 255.21) | 1631 (721 to 2955) | 192.75 (88.03 to 345.59) | 0.82 (0.77 to 0.88) |
| Guyana | 967 (548 to 1419) | 231.12 (129.37 to 348.17) | 3757 (2071 to 5808) | 549.65 (306.51 to 855.52) | 3.88 (3.52 to 4.23) |
| Haiti | 3127 (1370 to 5837) | 86.36 (38.12 to 158.66) | 12780 (5227 to 26857) | 148.95 (61.61 to 315.09) | 2.17 (2.03 to 2.3) |
| Honduras | 1926 (978 to 3067) | 90.36 (45.83 to 142.49) | 12364 (6038 to 19654) | 190.67 (92.39 to 308.34) | 2.68 (2.51 to 2.85) |
| Hungary | 8914 (4979 to 13209) | 63.68 (35 to 95.01) | 16017 (8296 to 24494) | 82.51 (42.92 to 126.58) | 1.43 (1.2 to 1.65) |
| Iceland | 102 (57 to 149) | 34.62 (19.17 to 50.25) | 304 (165 to 442) | 49.58 (27.24 to 72.26) | 1.32 (1.23 to 1.4) |
| India | 148738 (72048 to 251785) | 29.44 (14.22 to 49.36) | 838678 (423437 to 1361373) | 67.6 (33.91 to 108.54) | 2.81 (2.76 to 2.86) |
| Indonesia | 44127 (19936 to 76039) | 40.12 (17.63 to 69) | 237871 (109018 to 390159) | 88.14 (40.45 to 146.14) | 2.79 (2.64 to 2.93) |
| Iran (Islamic Republic of) | 23261 (11908 to 37011) | 90.48 (45.29 to 143.19) | 118152 (62523 to 175045) | 154.93 (80.87 to 230.66) | 1.81 (1.71 to 1.91) |
| Iraq | 26910 (15221 to 40226) | 326.06 (183.87 to 487.74) | 85118 (41355 to 134695) | 362.31 (179.21 to 589.43) | 0.01 (-0.12 to 0.15) |
| Ireland | 2536 (1259 to 3845) | 63.83 (31.42 to 96.66) | 6073 (3310 to 8959) | 75.5 (41.33 to 111.69) | 0.81 (0.69 to 0.92) |
| Israel | 5831 (3032 to 8703) | 124.65 (64.71 to 185.06) | 14938 (7336 to 22942) | 114.38 (56.32 to 175.57) | 0.12 (-0.24 to 0.49) |
| Italy | 48997 (24766 to 73620) | 55.88 (27.86 to 84.05) | 99727 (49077 to 151090) | 58.17 (28.76 to 87.46) | -0.01 (-0.11 to 0.09) |
| Jamaica | 2317 (1138 to 3630) | 131.57 (64.15 to 206.34) | 8680 (4564 to 13133) | 277.74 (146.63 to 420.8) | 2.03 (1.44 to 2.63) |
| Japan | 75752 (37628 to 122971) | 46.08 (22.8 to 74.84) | 181514 (87201 to 291777) | 44.93 (22.21 to 71.38) | -0.11 (-0.2 to -0.01) |
| Jordan | 3254 (1828 to 4778) | 247.6 (134.85 to 371.07) | 20905 (12663 to 29795) | 300.64 (178.46 to 429.76) | 0.39 (0.08 to 0.71) |
| Kazakhstan | 9742 (4952 to 14834) | 74.42 (38.19 to 113.01) | 21805 (11167 to 33256) | 120.87 (61.63 to 185.15) | 1.33 (1.08 to 1.58) |
| Kenya | 4778 (2228 to 8365) | 56.76 (27.08 to 100.86) | 34967 (17243 to 55849) | 147.67 (71.76 to 240.66) | 3.4 (3.31 to 3.5) |
| Kiribati | 80 (37 to 131) | 201.94 (91.31 to 337.92) | 270 (118 to 489) | 354.12 (154.06 to 645.52) | 1.7 (1.47 to 1.92) |
| Kuwait | 1489 (839 to 2172) | 216.03 (116.78 to 326.55) | 4824 (2961 to 6756) | 161.29 (95.13 to 228.32) | -0.76 (-0.98 to -0.54) |
| Kyrgyzstan | 2008 (1102 to 3051) | 64.46 (35.78 to 97.13) | 5857 (3279 to 9050) | 111.89 (61.66 to 169.65) | 1.07 (0.56 to 1.57) |
| Lao People's Democratic Republic | 1776 (805 to 3225) | 81.48 (36.39 to 149.41) | 5782 (2306 to 11230) | 115.15 (45.57 to 218.46) | 1.18 (1.12 to 1.24) |
| Latvia | 1407 (758 to 2143) | 40.8 (21.96 to 62.65) | 3115 (1723 to 4663) | 80.58 (43.87 to 122.46) | 2.2 (2.01 to 2.39) |
| Lebanon | 4221 (2224 to 6925) | 199.4 (102.97 to 325.65) | 15516 (8553 to 23937) | 248.5 (137.64 to 380.07) | 0.95 (0.76 to 1.14) |
| Lesotho | 682 (325 to 1118) | 81.96 (39.27 to 135.1) | 3186 (1448 to 5446) | 291.25 (130.86 to 498.39) | 5.1 (4.58 to 5.61) |
| Liberia | 2440 (1258 to 4133) | 201.61 (104.71 to 333.13) | 7864 (3911 to 12831) | 306.7 (151.25 to 514.66) | 1.63 (1.31 to 1.94) |
| Libya | 3160 (1635 to 5145) | 162.85 (83.29 to 267.56) | 22461 (10893 to 35516) | 414.13 (198.82 to 659.56) | 3.69 (3.47 to 3.9) |
| Lithuania | 1591 (839 to 2474) | 36.2 (18.88 to 56.32) | 3744 (1998 to 5506) | 67.49 (35.44 to 100.02) | 1.63 (1.44 to 1.82) |
| Luxembourg | 363 (196 to 532) | 68.45 (37.03 to 100.34) | 890 (434 to 1397) | 78.76 (38.91 to 121.77) | 0.73 (0.56 to 0.89) |
| Madagascar | 2017 (883 to 3865) | 40.17 (17.68 to 75.66) | 8233 (3442 to 14739) | 73.73 (30.81 to 131.91) | 1.98 (1.85 to 2.11) |
| Malawi | 3006 (1367 to 5641) | 76.96 (34.71 to 143.54) | 11452 (5397 to 19873) | 149.54 (71.46 to 256.18) | 1.95 (1.72 to 2.19) |
| Malaysia | 11732 (5966 to 18815) | 119.62 (61.38 to 189.72) | 59019 (30263 to 91523) | 202.47 (104.62 to 314.1) | 1.54 (1.38 to 1.7) |
| Maldives | 156 (68 to 267) | 147.6 (64.27 to 256.44) | 476 (219 to 807) | 119.78 (54.9 to 203.95) | -0.98 (-1.17 to -0.78) |
| Mali | 5118 (2469 to 8629) | 123.67 (60.29 to 210.61) | 14229 (7387 to 23936) | 148.01 (76.38 to 252.56) | 0.62 (0.46 to 0.77) |
| Malta | 266 (130 to 407) | 65.04 (31.95 to 99.2) | 808 (379 to 1262) | 80.74 (38.22 to 124.65) | 0.68 (0.44 to 0.93) |
| Marshall Islands | 36 (15 to 69) | 206.25 (85.39 to 410.03) | 150 (39 to 376) | 391.8 (99.74 to 1007.25) | 2.04 (1.82 to 2.26) |
| Mauritania | 2069 (1018 to 3237) | 211.62 (104.12 to 333.66) | 5771 (2900 to 9593) | 275.9 (135.92 to 446.62) | 0.51 (0.36 to 0.67) |
| Mauritius | 1466 (713 to 2394) | 190.34 (92.85 to 311.92) | 9634 (4834 to 15444) | 518.93 (257.87 to 830.5) | 3.52 (3.09 to 3.95) |
| Mexico | 103787 (55042 to 157719) | 237.77 (126.64 to 365.9) | 673923 (392964 to 965604) | 517.36 (301.28 to 743.75) | 3.02 (2.34 to 3.71) |
| Micronesia (Federated States of) | 120 (58 to 211) | 239.41 (114.59 to 431.24) | 367 (161 to 607) | 456.86 (191.81 to 770.66) | 2.12 (1.79 to 2.45) |
| Monaco | 40 (23 to 60) | 55.07 (31.72 to 81.79) | 84 (45 to 123) | 79.98 (44.18 to 116.61) | 1.32 (1.17 to 1.48) |
| Mongolia | 1344 (724 to 2078) | 118.81 (64.45 to 182.6) | 3407 (1838 to 5342) | 131.64 (71.64 to 203.13) | 0.05 (-0.07 to 0.18) |
| Montenegro | 715 (409 to 1076) | 115.56 (66.18 to 173.5) | 1513 (819 to 2243) | 164.74 (88.41 to 245.29) | 1.23 (1.11 to 1.35) |
| Morocco | 20462 (10717 to 35503) | 141.49 (73.85 to 249.51) | 93743 (49431 to 149294) | 274.49 (144.92 to 439.83) | 2.43 (2.32 to 2.55) |
| Mozambique | 2500 (1011 to 4460) | 40.32 (16.27 to 71.11) | 11787 (5149 to 21472) | 95.33 (40.85 to 177.07) | 3.24 (3.1 to 3.38) |
| Myanmar | 16191 (6808 to 28752) | 63.98 (27.27 to 112.57) | 41079 (18889 to 72979) | 79.24 (36.62 to 139.15) | 0.51 (0.43 to 0.6) |
| Namibia | 627 (296 to 1070) | 96.35 (45.1 to 164.14) | 2678 (1359 to 4410) | 192.41 (97.04 to 309.32) | 2.02 (1.68 to 2.36) |
| Nauru | 15 (7 to 27) | 294.22 (139.36 to 509.62) | 35 (17 to 53) | 559.73 (260.04 to 900.85) | 1.94 (1.81 to 2.07) |
| Nepal | 3742 (1609 to 6561) | 34.71 (15.07 to 59.48) | 19568 (8985 to 33500) | 77.78 (36.05 to 132.41) | 2.67 (2.39 to 2.95) |
| Netherlands | 8708 (4118 to 13310) | 43.63 (20.55 to 66.7) | 24823 (12448 to 37094) | 67.33 (33.8 to 100.15) | 1.47 (1.25 to 1.69) |
| New Zealand | 2259 (1253 to 3291) | 59.42 (32.87 to 86.76) | 7581 (4387 to 10674) | 90.51 (52.5 to 127.46) | 1.57 (1.23 to 1.91) |
| Nicaragua | 3322 (1654 to 5174) | 200.73 (98.92 to 312.37) | 25135 (13188 to 40013) | 484.11 (252.3 to 774.36) | 3.39 (3 to 3.78) |
| Niger | 2327 (1110 to 3991) | 76.85 (35.83 to 134.78) | 7556 (3447 to 13124) | 84.5 (39.08 to 147.77) | 0.17 (0.06 to 0.29) |
| Nigeria | 43300 (20839 to 72318) | 100.22 (48.05 to 165.19) | 163395 (81568 to 266095) | 174.53 (87.23 to 280.01) | 1.68 (1.59 to 1.77) |
| Niue | 5 (2 to 8) | 212.64 (99.46 to 351.63) | 10 (4 to 18) | 469.78 (195.43 to 844.95) | 2.54 (2.37 to 2.71) |
| North Macedonia | 1725 (916 to 2641) | 92.74 (49.37 to 143.17) | 3700 (1870 to 5730) | 119.34 (60.85 to 183.98) | 0.86 (0.65 to 1.07) |
| Northern Mariana Islands | 54 (24 to 85) | 285.1 (129.12 to 470.67) | 257 (114 to 398) | 490.44 (217.88 to 772.05) | 1.96 (1.78 to 2.14) |
| Norway | 2388 (1227 to 3655) | 35.08 (18.04 to 53.77) | 4988 (2600 to 7490) | 46.97 (24.42 to 70.46) | 1.07 (0.93 to 1.21) |
| Oman | 874 (409 to 1484) | 120.42 (54.91 to 199.36) | 7497 (4494 to 11199) | 362.05 (209.96 to 540.45) | 4.33 (4.07 to 4.58) |
| Pakistan | 41147 (20124 to 68430) | 69.85 (34.13 to 117.15) | 216552 (104173 to 362496) | 154.49 (72.49 to 260.34) | 2.56 (2.34 to 2.78) |
| Palau | 22 (11 to 37) | 228 (113.92 to 378.92) | 95 (45 to 151) | 427.14 (204.4 to 687.95) | 2.18 (1.99 to 2.36) |
| Palestine | 2281 (1239 to 3652) | 272.94 (145.84 to 445.3) | 7907 (4479 to 11831) | 328.54 (176.65 to 496.52) | 0.53 (0.34 to 0.71) |
| Panama | 1731 (913 to 2553) | 116.98 (62.09 to 171.11) | 12452 (6756 to 18346) | 280.27 (152.03 to 412.75) | 3.1 (2.74 to 3.47) |
| Papua New Guinea | 957 (382 to 1834) | 46.41 (18.77 to 89.76) | 3502 (1364 to 6586) | 57.67 (23.43 to 110.84) | 0.6 (0.54 to 0.66) |
| Paraguay | 3287 (1857 to 4775) | 146.73 (81.83 to 212.7) | 14747 (8040 to 21735) | 249.97 (135.17 to 369.66) | 1.85 (1.74 to 1.97) |
| Peru | 21000 (10850 to 30280) | 175.2 (89.54 to 255.23) | 86721 (48392 to 128306) | 256.89 (142.45 to 384.35) | 1.02 (0.83 to 1.22) |
| Philippines | 21407 (9663 to 36895) | 67.28 (30.17 to 116.93) | 141259 (60309 to 246424) | 158.86 (66.99 to 274.38) | 3.21 (3.03 to 3.39) |
| Poland | 43585 (23725 to 64620) | 101.48 (54.82 to 151.42) | 45940 (25901 to 70098) | 65.1 (36.89 to 99.4) | -1.5 (-1.89 to -1.11) |
| Portugal | 10202 (5445 to 15546) | 77.43 (41.16 to 119.32) | 24005 (12346 to 37109) | 87.16 (46.05 to 132.64) | 0.46 (0.05 to 0.88) |
| Puerto Rico | 9737 (5823 to 13929) | 275.11 (164.34 to 392.77) | 22862 (13591 to 33424) | 343.36 (209.3 to 487.61) | 1.36 (1.01 to 1.71) |
| Qatar | 286 (143 to 484) | 251.27 (122.5 to 447.02) | 3116 (1928 to 4450) | 357.49 (216.84 to 516.58) | 0.84 (0.29 to 1.4) |
| Republic of Korea | 13318 (6126 to 22851) | 49.49 (22.15 to 85.17) | 38495 (18372 to 64832) | 41.46 (19.82 to 69.7) | -0.31 (-0.45 to -0.17) |
| Republic of Moldova | 1884 (1045 to 2922) | 44.45 (24.26 to 68.71) | 4520 (2655 to 6726) | 77.43 (45.77 to 115.61) | 1.6 (1.45 to 1.76) |
| Romania | 24564 (13693 to 36766) | 91.93 (51.23 to 138.45) | 36202 (21623 to 52574) | 102 (60.53 to 148.11) | 0.74 (0.44 to 1.05) |
| Russian Federation | 79384 (41550 to 120343) | 45.42 (23.66 to 69.13) | 140722 (77923 to 204665) | 59.79 (33.33 to 87.44) | 0.41 (0.21 to 0.62) |
| Rwanda | 2079 (829 to 3919) | 70.15 (29.37 to 134.07) | 5312 (2178 to 9946) | 83.3 (33.83 to 156.24) | -0.38 (-0.77 to 0.02) |
| Saint Kitts and Nevis | 110 (60 to 170) | 319.78 (178.17 to 475.69) | 366 (205 to 531) | 517.71 (289.91 to 751.22) | 2.35 (2.05 to 2.66) |
| Saint Lucia | 193 (100 to 290) | 222.71 (116.67 to 336.21) | 815 (441 to 1232) | 342.3 (185.13 to 513.73) | 1.86 (1.6 to 2.11) |
| Saint Vincent and the Grenadines | 99 (48 to 159) | 139.97 (68.68 to 223.55) | 438 (234 to 671) | 314.74 (169.65 to 483.75) | 3.28 (2.99 to 3.58) |
| Samoa | 212 (101 to 349) | 239.44 (112.62 to 399.72) | 599 (289 to 912) | 406.79 (193.53 to 625.24) | 1.71 (1.64 to 1.78) |
| San Marino | 17 (9 to 25) | 47.06 (25.95 to 69.1) | 39 (21 to 61) | 47.46 (25.36 to 71.98) | 0.59 (0.4 to 0.77) |
| Sao Tome and Principe | 130 (65 to 205) | 204.94 (101.5 to 323.5) | 495 (249 to 779) | 425.18 (210.62 to 659.72) | 2.27 (2.16 to 2.38) |
| Saudi Arabia | 19530 (10277 to 31223) | 307.6 (158.41 to 501.4) | 170261 (100966 to 245817) | 764.26 (444.94 to 1126.37) | 2.69 (2.47 to 2.91) |
| Senegal | 5125 (2562 to 8213) | 153.2 (76.21 to 245.87) | 16014 (7889 to 27119) | 198.51 (95.51 to 333.45) | 0.65 (0.53 to 0.77) |
| Serbia | 10962 (5826 to 17197) | 105.04 (56.6 to 165) | 20786 (11038 to 32090) | 126.44 (67.24 to 193.75) | 0.5 (0.41 to 0.6) |
| Seychelles | 99 (51 to 154) | 175.73 (91.53 to 273.35) | 422 (212 to 666) | 352.83 (173.92 to 553.45) | 2.41 (2.2 to 2.63) |
| Sierra Leone | 1788 (841 to 3105) | 87.72 (41.99 to 151.77) | 4926 (2400 to 8317) | 121.2 (59.72 to 204.73) | 1.01 (0.98 to 1.04) |
| Singapore | 971 (472 to 1604) | 44.4 (21.46 to 73.75) | 4244 (2029 to 6647) | 49.82 (23.81 to 78.76) | 1.55 (1.13 to 1.97) |
| Slovakia | 6102 (3538 to 9041) | 103.11 (59.36 to 152.17) | 8702 (4968 to 12399) | 93.27 (53.51 to 133.81) | -0.33 (-0.37 to -0.29) |
| Slovenia | 1508 (832 to 2254) | 62.18 (34.29 to 93.6) | 2947 (1636 to 4319) | 64.16 (35.82 to 94.15) | 0.27 (0.12 to 0.42) |
| Solomon Islands | 172 (61 to 364) | 110.23 (41.53 to 232.72) | 507 (185 to 972) | 125.02 (45.26 to 244.13) | 0.15 (-0.04 to 0.34) |
| Somalia | 2052 (899 to 4065) | 76.3 (34.68 to 149.79) | 8345 (3675 to 15756) | 120.85 (53.01 to 226.24) | 1.57 (1.5 to 1.65) |
| South Africa | 28089 (14580 to 43834) | 125.35 (64.93 to 196.6) | 130631 (70383 to 194853) | 276.15 (147.09 to 415.81) | 2.48 (2.12 to 2.84) |
| South Sudan | 1347 (598 to 2803) | 52.6 (23.25 to 109.42) | 4146 (1716 to 7809) | 103 (42.7 to 199.82) | 2.11 (1.85 to 2.36) |
| Spain | 41260 (20801 to 62967) | 77.31 (38.91 to 118.16) | 78900 (38166 to 122752) | 68.56 (34.56 to 104.01) | -0.18 (-0.28 to -0.08) |
| Sri Lanka | 6817 (2948 to 12160) | 60.77 (26.54 to 107.28) | 24231 (10145 to 45405) | 90.41 (37.62 to 170.67) | 1.22 (1.01 to 1.42) |
| Sudan | 11972 (5963 to 21048) | 124.45 (61.63 to 219.5) | 51229 (24864 to 83497) | 244.5 (116.84 to 407.58) | 2.24 (2.06 to 2.42) |
| Suriname | 500 (263 to 765) | 181.52 (95.09 to 282.67) | 2205 (1130 to 3553) | 340.88 (174.79 to 548.99) | 2.29 (2.1 to 2.47) |
| Sweden | 4814 (2434 to 7317) | 31.26 (15.82 to 47.72) | 12465 (6275 to 19485) | 50.76 (25.83 to 78.56) | 1.98 (1.85 to 2.1) |
| Switzerland | 4901 (2361 to 7614) | 45.96 (22.17 to 71.11) | 10703 (4782 to 17843) | 50.78 (23.12 to 83.49) | 0.82 (0.62 to 1.01) |
| Syrian Arab Republic | 13389 (7286 to 21213) | 252.82 (137.03 to 402.48) | 51095 (28678 to 78861) | 409.14 (221.46 to 633.81) | 1.18 (0.9 to 1.45) |
| Taiwan (Province of China) | 15137 (6775 to 26061) | 105.64 (47.11 to 183.58) | 51722 (23461 to 86673) | 119.57 (54.57 to 200.62) | 0.97 (0.72 to 1.22) |
| Tajikistan | 1329 (723 to 2070) | 46.88 (25.37 to 72.78) | 3842 (2077 to 5953) | 60.84 (33.01 to 94.07) | 0.7 (0.62 to 0.79) |
| Thailand | 22564 (9623 to 40197) | 60.9 (26.36 to 109.19) | 154844 (67202 to 273661) | 143.12 (61.96 to 251.58) | 2.47 (2.26 to 2.69) |
| Timor-Leste | 92 (36 to 179) | 29.74 (11.84 to 57.52) | 435 (182 to 863) | 49.67 (20.93 to 100.34) | 1.95 (1.62 to 2.29) |
| Togo | 1259 (616 to 2078) | 100.04 (48.59 to 167.28) | 6471 (3169 to 11121) | 169.15 (82.37 to 281.25) | 1.55 (1.48 to 1.62) |
| Tokelau | 2 (1 to 4) | 156.92 (73.04 to 291.53) | 4 (2 to 7) | 288.17 (129.3 to 474.98) | 2 (1.86 to 2.15) |
| Tonga | 76 (35 to 122) | 129.89 (58.98 to 213.29) | 181 (82 to 276) | 224.72 (100.25 to 343.51) | 1.62 (1.46 to 1.79) |
| Trinidad and Tobago | 1533 (824 to 2236) | 178.47 (95.2 to 263.35) | 7867 (4236 to 12355) | 409.82 (221.06 to 640.43) | 3.22 (2.9 to 3.55) |
| Tunisia | 4819 (2407 to 7913) | 96.19 (47.29 to 160.26) | 26998 (13421 to 43497) | 208.96 (104.08 to 338.12) | 2.57 (2.54 to 2.6) |
| Türkiye | 68105 (37505 to 105726) | 203.36 (109.72 to 317.44) | 217896 (122907 to 323605) | 239.13 (134.14 to 356.89) | 0.88 (0.57 to 1.19) |
| Turkmenistan | 1983 (1049 to 3108) | 94.27 (50.09 to 144.08) | 8118 (4348 to 13273) | 182.4 (96.88 to 297.58) | 2.05 (1.72 to 2.39) |
| Tuvalu | 11 (4 to 20) | 146.72 (57.88 to 282.3) | 30 (11 to 55) | 282.43 (108.11 to 518.4) | 2.18 (2.09 to 2.26) |
| Uganda | 3631 (1535 to 6695) | 54.81 (23.33 to 101.41) | 16122 (7248 to 29425) | 102.61 (45.94 to 188.32) | 1.63 (1.43 to 1.83) |
| Ukraine | 15799 (8610 to 24421) | 23.62 (12.82 to 36.4) | 33513 (18340 to 51044) | 46.23 (25.11 to 71.14) | 2.64 (2.38 to 2.91) |
| United Arab Emirates | 957 (542 to 1585) | 159.13 (81.78 to 268.11) | 12556 (7186 to 17621) | 356.88 (184.27 to 506.28) | 4.48 (3.89 to 5.07) |
| United Kingdom | 37231 (17924 to 56602) | 42.52 (20.48 to 65.05) | 67528 (32798 to 105294) | 51.34 (25.56 to 79.66) | 0.86 (0.75 to 0.97) |
| United Republic of Tanzania | 10136 (5049 to 16938) | 92.27 (44.26 to 154.52) | 45260 (23394 to 76212) | 170.05 (88.62 to 285.34) | 2 (1.95 to 2.04) |
| United States of America | 265590 (145681 to 369143) | 84.53 (46.77 to 116.93) | 1286175 (742874 to 1769939) | 226.76 (134.12 to 307.14) | 3.52 (3.32 to 3.73) |
| United States Virgin Islands | 182 (108 to 254) | 204.02 (118.22 to 291.51) | 378 (210 to 568) | 245.18 (142.13 to 357.24) | 0.97 (0.75 to 1.18) |
| Uruguay | 3111 (1525 to 4593) | 80.04 (39.08 to 117.42) | 6222 (3060 to 9450) | 108.27 (54.65 to 161.37) | 1.24 (1.02 to 1.46) |
| Uzbekistan | 7741 (4165 to 11673) | 66.03 (35.37 to 100.84) | 42191 (21353 to 65353) | 146.4 (74.22 to 228.21) | 2.17 (1.7 to 2.64) |
| Vanuatu | 88 (41 to 167) | 135.95 (63.19 to 256.33) | 412 (183 to 748) | 218.27 (96.26 to 401.52) | 1.47 (1.41 to 1.52) |
| Venezuela (Bolivarian Republic of) | 14940 (8097 to 22198) | 146.46 (79.1 to 216.46) | 108473 (59122 to 166724) | 356.9 (192.53 to 548.26) | 2.51 (2.11 to 2.91) |
| Viet Nam | 9705 (4159 to 18713) | 24.22 (10.31 to 46.64) | 50039 (20022 to 93454) | 49.54 (19.57 to 91.32) | 2.84 (2.6 to 3.08) |
| Yemen | 3766 (1827 to 6989) | 75.13 (36.69 to 143.47) | 17781 (8734 to 30816) | 124.16 (60.27 to 219.31) | 1.54 (1.35 to 1.73) |
| Zambia | 2883 (1330 to 5113) | 98.06 (44.82 to 173.57) | 16186 (7627 to 29905) | 210.32 (100.54 to 373.56) | 2.12 (1.95 to 2.3) |
| Zimbabwe | 3099 (1384 to 5306) | 74.79 (33.83 to 128.29) | 16619 (7732 to 28424) | 224.68 (103.57 to 381.46) | 4.03 (3.45 to 4.62) |

Abbreviations: DALY, disability-adjusted life year; BMI, body mass index; ASDR, age-standardized DALYs rate; EAPC, estimated annual percentage change.

**
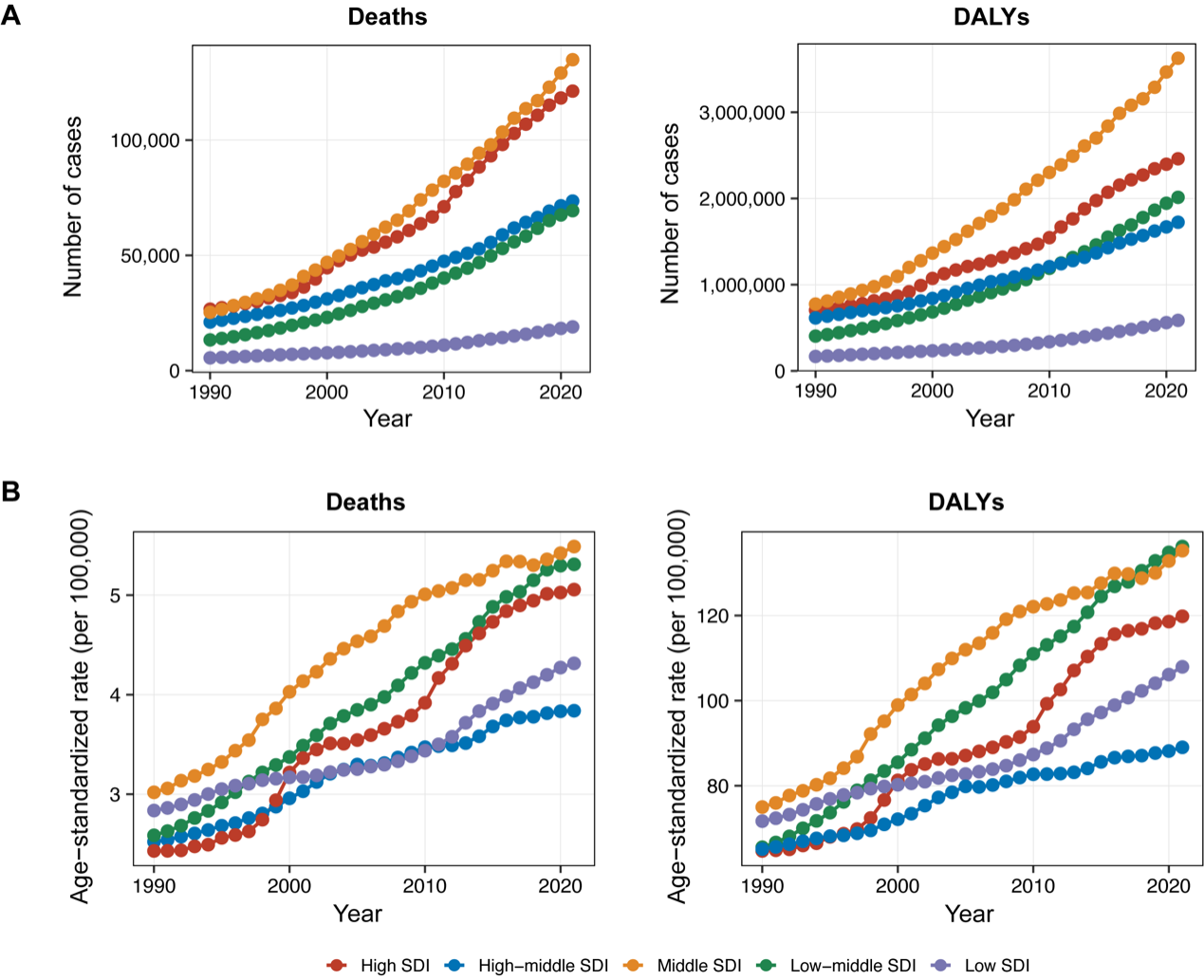
**

**Supplementary Figure 1.** The global disease burden of CKD attributable to high BMI by SDI. (A) Numbers of deaths and DALYs. (B) Age-standardized rates of deaths and DALYs.


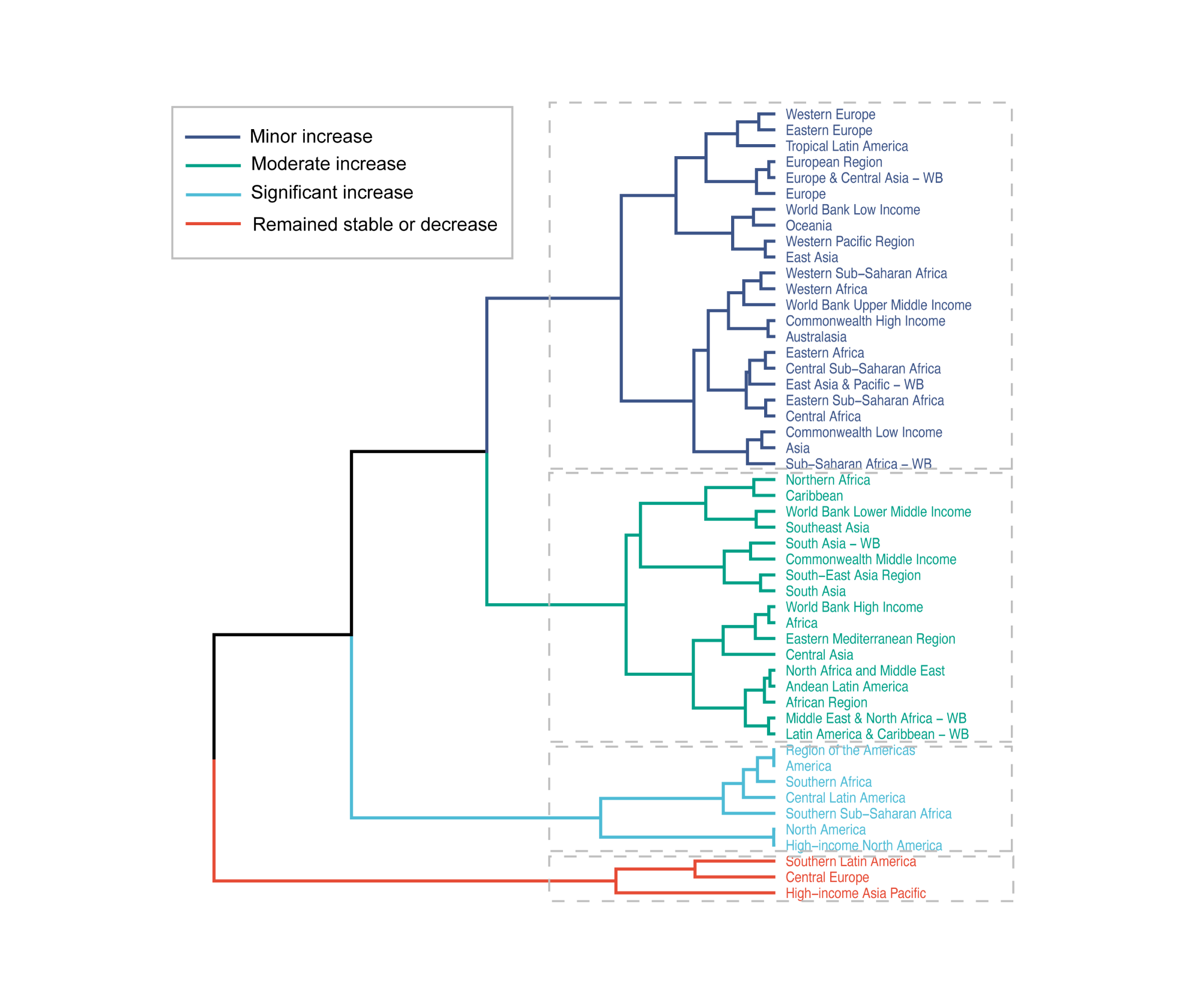


**Supplementary Figure 2.** Results of cluster analysis based on the EAPC values for CKD-related ASMRs and ASDRs attributable to high BMI, from 1990 to 2021. Abbreviations: EAPC, estimated annual percentage change; CKD, chronic kidney disease; ASMR, age-standardized mortality rate; ASDR, age-standardized disability-adjusted life years rate; BMI, body mass index.
